# Cross-talk between glycosylation pathways: Mechanistic insights and implications for human diseases

**DOI:** 10.1016/j.molmet.2026.102400

**Published:** 2026-06-13

**Authors:** Ninon Very, Ikram El Yazidi-Belkoura

**Affiliations:** Université de Lille, CNRS, UMR8576 UGSF - Unité de Glycobiologie Structurale et Fonctionnelle, F-59000, Lille, France

**Keywords:** Glycosylation network, UDP-GlcNAc, Metabolic regulation, *O*-GlcNAcylation, Human diseases

## Abstract

Glycosylation encompasses a broad spectrum of post-translational modifications (PTMs) that shape protein stability, spatial organization, and function. Traditionally, it is classified into two major categories: complex glycosylation within the secretory pathway — including *N*-glycosylation, mucin-type *O*-glycosylation, glycosaminoglycans (GAGs), and glycolipids — which generate structurally stable and long-lived modifications; and *O*-GlcNAcylation, a highly dynamic modification of nucleocytoplasmic and mitochondrial proteins that rapidly responds to metabolic and environmental cues. While this dichotomous framework has guided our understanding of glycan biology, emerging evidence now reveals that glycosylations are functionally interconnected through shared metabolic substrates, and regulatory circuits.

Here, we revisit this classical classification and integrate it into a modern, systems-level view of glycosylation. We highlight the nucleotide sugar UDP-N-acetylglucosamine (UDP-GlcNAc) as a metabolic node reflecting cellular nutrient status and fuelling both complex glycan synthesis and *O*-GlcNAcylation. UDP-GlcNAc pool fluctuations drive coordinated remodeling across glycosylation pathways, and dysregulation of this hub is associated with diverse human diseases. We discuss how *O*-GlcNAcylation functions as a supplementary regulatory PTM, modulating glycosylation-related enzymes and proteins through both direct effects on their interactions, stability, localisation and activity, and *via* broader transcriptional and epigenetic programs, thereby dynamically controlling otherwise stable glycosylation processes. Examples from metabolic, cardiovascular, neurological diseases, cancer and congenital disorders of glycosylation (CDGs) illustrate how perturbations in one glycosylation pathway propagate through the glycosylation network, reshaping cellular identity and disease trajectories. We support a paradigm in which glycosylation operates as an integrated regulatory framework linking metabolism, signaling, and extracellular architecture, providing new perspectives for disease stratification and therapeutic intervention.

## Highlights

• New paradigm in which glycosylations operate as an integrated regulatory framework.
• UDP-GlcNAc is a central hub linking metabolism to multiple glycosylation pathways.
• Glycosylation crosstalk shapes disease-specific cellular functions.
• *O*-GlcNAcylation regulates multiple components of the glycosylation machinery.
• Targeting HBP enzymes or *O*-GlcNAc cycling enables precision therapy.

## 1 Introduction

**Figure 1:**
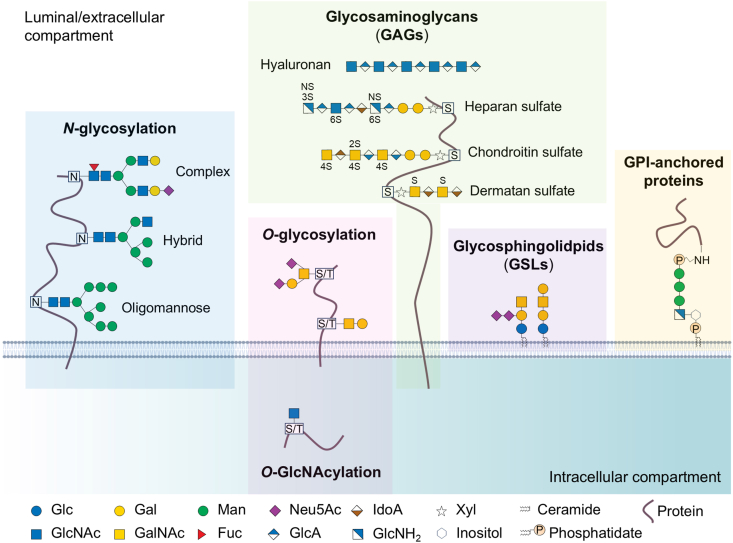
**Major classes of glycosylation in human cells.** Protein glycosylation involves the covalent attachment of carbohydrate moieties to specific amino acid residues. *N*-glycans are linked *via* N-acetylglucosamine (GlcNAc) to asparagine (Asn, N) residues and can adopt oligomannose, hybrid, or complex structures. *O*-glycans are initiated by N-acetylgalactosamine (GalNAc) attached to serine (Ser, S) or threonine (Thr, T) residues, and both *N*- and *O*-glycans can be further elongated and modified, commonly through fucosylation and sialylation. *O-*GlcNAcylation refers to the addition of a single N-acetylglucosamine to S/T residues of intracellular proteins in the cytoplasm, nucleus, or mitochondria. Other glycoconjugates synthesized in the lumen of the secretory pathway include glycosaminoglycans (GAGs), glycosphingolipids (GSLs), and glycosylphosphatidylinositol (GPI) anchors. Most GAG chains are covalently attached to S residues of core proteins *via* a tetrasaccharide linker composed of xylose (Xyl)–galactose (Gal)–Gal–glucuronic acid (GlcA) to form proteoglycans (e.g.*,* heparan sulfate, chondroitin sulfate, dermatan sulfate), whereas hyaluronan is synthesized as a free, non–protein-linked polymer at the cell surface. GSLs, consisting of a ceramide lipid linked to oligosaccharides, can be further modified with terminal sialic acids to form gangliosides. GPI anchors attach proteins to the extracellular leaflet of the plasma membrane *via* a phosphatidate lipid, linking a glycan core to the membrane. Fuc, fucose; Gal, galactose; GalNAc, N-acetylgalactosamine; Glc, glucose; GlcA, glucuronic acid; GlcNH_2_, glucosamine; GlcNAc, N-acetylglucosamine; IdoA, iduronic acid; Man, mannose; Neu5Ac, N-acetylneuraminic acid; Xyl, xylose.

Glycosylation represents one of the most abundant and structurally diverse post-translational modifications (PTMs) found across all eukaryotic organisms [1]. It consists of the covalent, enzymatic-mediated attachment of carbohydrates — referred to as glycans — to proteins (glycoproteins), lipids (glycolipids), or other biomolecules (Figure 1). In human, this highly coordinated biosynthetic network involves approximatively 700 genes encoding glycosyltransferases (GTs), glycosylhydrolases (GHs), transporters and chaperones [2] and the modification of more than 50% of all proteins [3], collectively producing a glycome whose informational complexity rivals that of the proteome. The three main types of glycosylation [4] are: (i) *N*-glycosylation, which begins in the *endoplasmic reticulum* (*ER*) with the transfer of a pre-assembled oligosaccharide (Glc_3_Man_9_GlcNAc_2_) onto asparagine (Asn) residues within the Asn-X-threonine (Thr)/serine (Ser) consensus sequon, followed by stepwise processing and maturation in the Golgi apparatus to generate high-mannose, hybrid, or complex structures; (ii) *O*-glycosylation, which primarily occurs in the Golgi, where N-acetylgalactosamine (GalNAc) is transferred to Ser or Thr residues by polypeptide N-acetylgalactosaminyltransferase (GALNT) enzymes, initiating mucin-type chains that can be extended into branched motifs; and (iii) *O*-GlcNAcylation, a dynamic nucleocytoplasmic or mitochondrial modification in which a single N-acetylglucosamine (GlcNAc) residue is added to Ser/Thr by *O*-GlcNAc transferase (OGT). Other significant forms include: (iv) glycosaminoglycans (GAGs), long unbranched polysaccharides — such as heparan sulfate (HS) or chondroitin sulfate (CS) — covalently linked to core proteins to form proteoglycans; (v) glycosphingolipids (GSLs), glycans attached to ceramide-based lipids in cellular membranes, contributing to glycocalyx formation; and (vi) glycosylphosphatidylinositol (GPI) anchoring, in which pre-assembled GPI anchors are post-translationally linked to C-terminal residues, tethering proteins to lipid rafts (Figure 1).

Glycosylation imparts distinct physicochemical properties that critically control protein folding, stability, trafficking and secretion [3,4]. *N*-glycans, for instance, interact with *ER* chaperones like calnexin and calreticulin to enforce protein quality control during co-translational folding, while shielding vulnerable sites to enhance solubility and proteolytic resistance [4]. Beyond these structural roles, glycans orchestrate a wide array of biological processes such as intracellular trafficking, cell–cell and cell–matrix interactions, signal transduction, host-pathogen recognition, and immune responses [5]. Distinct glycan classes specialize in particular functions: *N*-glycosylation stabilizes surface receptors and matures secreted proteins while *O*-glycosylation forms protective mucin barriers and fine-tunes receptor signaling. Concurrently, *O*-GlcNAcylation dynamically regulates transcription, metabolism, and stress responses. In the extracellular environment, GAGs reinforce the matrix and sequester growth factors, whereas GSLs scaffold membrane rafts to coordinate signaling and microbial sensing. Finally, GPI anchors proteins to lipid domains, optimizing trafficking and immune functions [4]. Together, these diverse mechanisms establish glycosylation as a pivotal regulator of cellular physiology and homeostasis. Consequently, when these pathways are perturbed, the resulting disruption of folding, trafficking, and signaling cascades can compromise multiple cellular functions simultaneously, laying the groundwork for a broad spectrum of human diseases.

## 2 UDP-GlcNAc: nutrient integrator in glycosylation, metabolism, and human diseases

**Figure 2:**
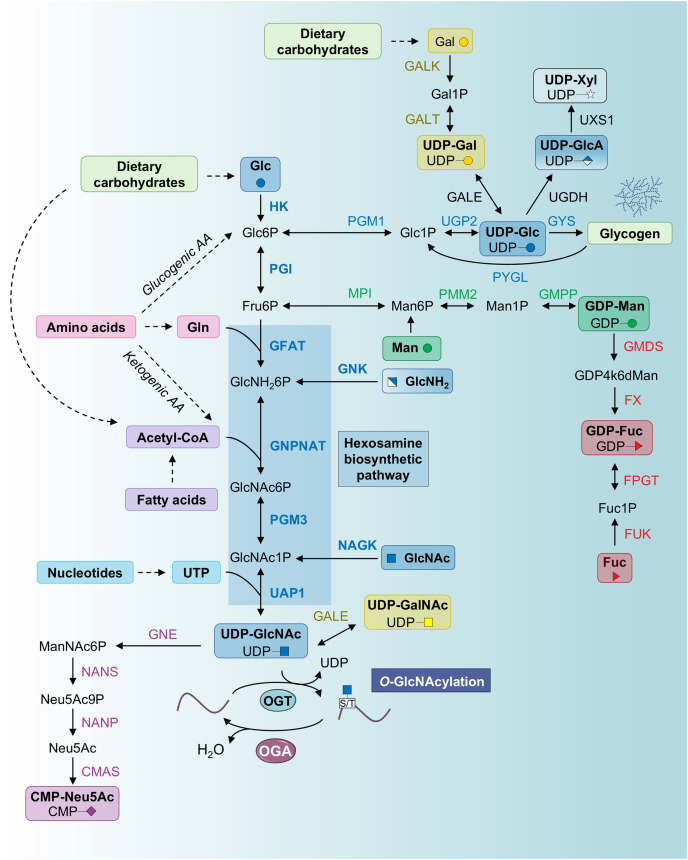
**Central role of UDP-GlcNAc in nucleotide sugar biosynthesis and *O*-GlcNAcylation *via* the hexosamine biosynthetic pathway (HBP).** Overview of metabolic pathways converging on nucleotide sugar synthesis. The hexosamine biosynthetic pathway (HBP) occupies a central position at the intersection of carbohydrate, amino acid, fatty acid, and nucleotide metabolisms, producing UDP-N-acetylglucosamine (UDP-GlcNAc), a key donor for *O*-GlcNAcylation and multiple other glycosylation reactions. Glucose (Glc) derived from dietary starch, other carbohydrates, or glycogen is first phosphorylated by Hexokinase (HK) to form glucose-6-phosphate (Glc6P), then isomerized by Phosphoglucose isomerase (PGI) to fructose-6-phosphate (Fru6P). The rate-limiting step of the HBP is catalyzed by Glutamine: fructose-6-phosphate amidotransferase (GFAT), which transfers an amino group from glutamine (Gln) to Fru6P to produce glucosamine-6-phosphate (GlcNH_2_6P). GlcNH_2_6P is then acetylated by Glucosamine-6-phosphate N-acetyltransferase (GNPNAT), using acetyl-coenzyme A (acetyl-CoA) as the acetyl donor, to form N-acetylglucosamine-6-phosphate (GlcNAc6P). This intermediate is converted into N-acetylglucosamine-1-phosphate (GlcNAc1P) by Phosphoglucomutase 3 (PGM3) and subsequently activated by UDP-N-acetylglucosamine pyrophosphorylase (UAP1) in a reaction consuming UTP, yielding UDP-GlcNAc. A salvage pathway *via* N-acetylglucosamine kinase (NAGK) phosphorylates free GlcNAc to GlcNAc6P, feeding back into the HBP, while glucosamine (GlcNH_2_) can also enter the pathway through its phosphorylation to GlcN6P by Glucosamine kinase (GNK). UDP-GlcNAc can be further converted into UDP-N-acetylgalactosamine (UDP-GalNAc) and also underlies the synthesis of CMP-sialic acid (CMP-Neu5Ac), although the latter branch additionally requires flux through the N-acetylmannosamine (ManNAc) and sialic acid pathway. In parallel, Glc6P and Fru6P are diverted into the *de novo* synthesis of other nucleotide sugars. Glc6P can generate UDP-glucose (UDP-Glc), which can be epimerized into UDP-galactose (UDP-Gal) or oxidized into UDP-glucuronic acid (UDP-GlcA), the latter serving as a precursor for UDP-xylose (UDP-Xyl). Fru6P can be converted into GDP-mannose (GDP-Man), which in turn is transformed into GDP-fucose (GDP-Fuc). These pathways can also incorporate exogenous monosaccharides, such as Man, Fuc or Gal, independently of carbohydrate flux through the HBP, to replenish the corresponding nucleotide sugar pools. UDP-GlcNAc provides the substrate for *O*-GlcNAc transferase (OGT) which dynamically attach *O*-GlcNAc on serine (Ser, S) and threonine (Thr, T) residues of nucleocytoplasmic or mitochondrial proteins, while *O*-GlcNAcase (OGA) removes it. AA, amino acids; CMAS, CMP-N-acetylneuraminic acid synthetase; FPGT, Fucose-1-phosphate guanylyltransferase; FUK, Fucokinase; FX, GDP-4-keto-6-deoxymannose 3,5-epimerase 4-reductase; GALE, UDP-galactose 4-epimerase; GALK, Galactokinase; GALT, Galactose-1-phosphate uridylyltransferase; GMDS, GDP-mannose 4,6-dehydratase; GMPP, Mannose-1-phosphate guanylyltransferase; GNE, UDP-N-acetylglucosamine 2-epimerase/N-acetylmannosamine kinase; GYS, Glycogen synthase; MPI, Mannose phosphate isomerase; NANP, N-acetylneuraminate-9-phosphatase; NANS, N-acetylneuraminate synthase; PGM1, Phosphoglucomutase 1; PMM2, Phosphomannomutase 2; UGDH, UDP-glucose 6-dehydrogenase; UGP2, UTP-glucose-1-phosphate uridylyltransferase 2; UXS1, UDP-xylose synthase 1.

The nucleotide sugar uridine diphosphate N-acetylglucosamine (UDP-GlcNAc) is the final product of the hexosamine biosynthetic pathway (HBP), a metabolic route positioned at the crossroads of carbohydrates, amino acids, lipids, nucleotides and energy homeostasis (Figure 2). As such, UDP-GlcNAc acts as a central node that reflects the overall metabolic state of the cell. Accordingly, the intracellular pool of UDP-GlcNAc serves as a highly sensitive indicator of nutrient availability. Conditions of high glucose or glucosamine availability lead to increased UDP-GlcNAc levels [6]. Conversely, glucose deprivation or pharmacological inhibition of the HBP rate-limiting enzyme Glutamine:fructose-6-phosphate amidotransferase (GFAT) results in a marked reduction of this pool [7]. UDP-GlcNAc serves as the essential substrate for OGT, enabling the nucleocytoplasmic or mitochondrial *O*-GlcNAcylation of proteins. Beyond this role, UDP-GlcNAc contributes also to complex glycosylation pathways through both direct use and metabolic conversion. In the cytosol, UDP-GlcNAc can be epimerized by UDP-galactose-4-epimerase (GALE) to form UDP-GalNAc, which serves as a key donor for the biosynthesis of mucin-type *O*-glycans, complex glycolipids, and proteoglycans. In parallel, UDP-GlcNAc is metabolized by the bifunctional UDP-N-acetylglucosamine 2-epimerase/N-acetylmannosamine kinase (GNE) to generate N-acetylmannosamine (ManNAc), thereby fueling the sialic acid biosynthetic pathway and the subsequent production of cytidine-5′-monophospho-N-acetylneuraminic acid (CMP-Neu5Ac), the activated donor required for terminal sialylation of glycoproteins and glycolipids. Through these interconnected routes, UDP-GlcNAc also indirectly contributes to the synthesis of other nucleotide sugars, including GDP-mannose (GDP-Man) and GDP-fucose (GDP-Fuc) (Figure 2), which are essential for *N*-glycan, glycolipid, proteoglycan and GAG assembly [8,9]. Collectively, these pathways position UDP-GlcNAc as a central metabolic hub supporting the biosynthesis of a wide range of complex glycoconjugates [10]. As a result, changes in UDP-GlcNAc abundance and availability directly impact multiple glycosylation processes, encompassing protein *O*-GlcNAcylation [11], branched *N-*glycan formation [12,13], mucin-type *O*-glycosylation [14], as well as GAG and GSL synthesis [15,16]. Consequently, any metabolic imbalance affecting the intracellular UDP-GlcNAc pool has the potential to trigger a coordinated deregulation of these glycosylation pathways.

**Figure 3:**
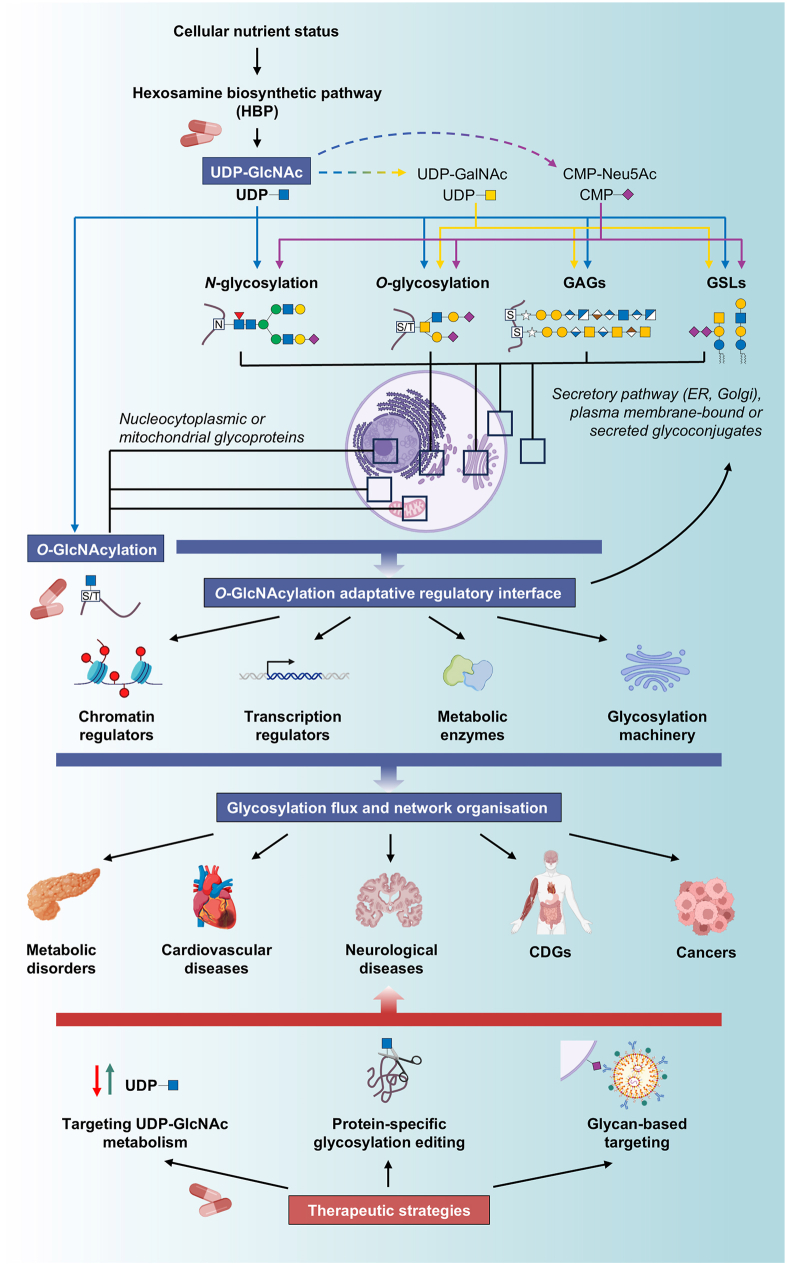
**Targeting the glyco-network: metabolic control of glycosylation in human diseases.** Cellular nutrient availability feeds into the hexosamine biosynthetic pathway (HBP), a metabolic sensor integrating carbohydrate, amino acid, nucleotide, and energy metabolisms to produce UDP-N-acetylglucosamine (UDP-GlcNAc). This central metabolite is a hub that is either directly used or converted into other nucleotide sugars, including UDP-N-acetylgalactosamine (UDP-GalNAc) and CMP-sialic acid (CMP-Neu5Ac), thereby supplying multiple glycosylation pathways. These encompass nucleocytoplasmic and mitochondrial *O*-GlcNAcylation, as well as *N*-glycosylation, *O*-glycosylation, glycosaminoglycan (GAG) biosynthesis, and glycosphingolipid (GSL) production within the secretory pathway. Beyond serving as a substrate, *O*-GlcNAcylation functions as an adaptive regulatory interface linking nutrient sensing to the organization of cellular glycosylation networks. As a dynamic and reversible post-translational modification, it modulates protein stability, localization, protein–protein interactions, and transcriptional or enzymatic activity, thereby regulating chromatin regulators, transcription factors, metabolic enzymes, and components of the glycosylation machinery. Through these mechanisms, *O*-GlcNAcylation coordinates glycosylation flux and enables extensive crosstalk between distinct glycosylation pathways, shaping an integrated cellular “glyco-network.” Perturbations in this regulatory axis propagate across intracellular signaling and extracellular interactions and are increasingly recognized as hallmarks of pathophysiological activation in mammalian cells, contributing to metabolic, cardiovascular and neurodegenerative diseases, cancers, and congenital disorders of glycosylation (CDGs). Therapeutic strategies thus seek to modulate this network at several levels. These include regulating HBP flux and UDP-GlcNAc production, pharmacologically targeting of *O*-GlcNAc cycling enzymes, editing protein-specific glycosylation, and leveraging disease-associated glycan signatures for diagnosis and targeted drug delivery. By intervening at this central metabolic node or addressing pathological glycan patterns, these approaches may reprogram dysregulated glycosylation networks and restore cellular homeostasis rather than merely targeting isolated downstream effectors.

Such coordinated deregulation of glycosylation pathways, resulting from alterations in UDP-GlcNAc metabolism, is increasingly recognized as central features of a wide range of pathologies, including metabolic disorders, congenital disorders of glycosylation (CDGs), cancer, neurodegenerative diseases, and immune dysfunctions [17] (Figure 3). These conditions highlight the sensitivity of cellular homeostasis to fluctuations in UDP-GlcNAc levels and its partitioning among competing glycosylation processes. A striking illustration of this vulnerability is provided by mutations in Phosphoglucomutase 3 (PGM3), which catalyzes the conversion of GlcNAc-6-phosphate (GlcNAc-6-P) to GlcNAc-1-phosphate (GlcNAc-1-P) at the third-step reaction of the HBP. *PGM3* mutations cause a severe form of CDG (PGM3-CDG), characterized by profound immunodeficiencies [18], notably affecting T and B cell functions. Mechanistically, impaired PGM3 activity leads to reduced UDP-GlcNAc levels, resulting in defective *N*-glycosylation of cell surface receptors, altered intracellular *O-*GlcNAcylation, and broad metabolic disturbances involving glycolysis and mitochondrial respiration. These combined defects ultimately compromise T-cell receptor (TCR)-mediated proliferation and immune competence [19]. Beyond rare genetic diseases, altered PGM3 expression has also been associated with more prevalent metabolic conditions, such as gestational diabetes mellitus [20], further underscoring the systemic impact of HBP dysregulation. Beyond absolute changes in UDP-GlcNAc production, growing evidence indicates that disease-associated phenotypes can arise from altered competition between UDP-GlcNAc–consuming glycosylation pathways. In the Alzheimer's disease brain, for instance, glycosylation homeostasis is profoundly disturbed, with an inverse relationship observed between protein *O*-GlcNAcylation and global *N*- and *O*-glycosylation levels [21]. In osteoarthritic cartilage, downregulation of UDP-GlcNAc transporters of the Solute carrier family 35 (SLC35) family at the *ER* and Golgi limits luminal substrate availability for GAG synthesis. As a consequence, UDP-GlcNAc accumulates in the cytosol, enhancing *O-*GlcNAcylation in chondrocytes and stabilizing transcription factors (TFs) such as GATA4, a regulator of stress-induced pro-inflammatory secretory programs associated with senescence, thereby contributing to disease progression [22]. These observations have led to the proposal that limited UDP-GlcNAc availability drives competition among glycosylation pathways, thereby reshaping cellular signaling and protein function.

Among these competing pathways, hyaluronan (HA) synthesis emerges as a major determinant of UDP-GlcNAc allocation. HA is a high-molecular weight GAG whose biosynthesis places a substantial demand on the cellular UDP-GlcNAc pool, allowing it to function as a metabolic rheostat that redistributes this key metabolite between extracellular matrix (ECM) production and intracellular glycosylation processes [23]. Conversely, recent work has demonstrated that dynamic regulation of HA turnover can also reprogram UDP-GlcNAc distribution in a metabolically beneficial manner. In postprandial hepatocytes, lysosomal Hyaluronidase-1 (HYAL1)-mediated degradation of circulating HA activates a feedback loop that stimulates HA resynthesis and redistributes subcellular UDP-GlcNAc pools, potentially towards the plasma membrane. This redistribution reduces mitochondrial *O*-GlcNAcylation, including the glycosylation of ATP synthase subunits, leading to decreased ATP production and suppression of hepatic gluconeogenesis. As a result, postprandial glucose levels are lowered, providing protection against insulin-resistant states such as type 2 diabetes (T2D) [24]. In sharp contrast, malignant contexts exploit enhanced UDP-GlcNAc availability to support tumor progression. Aggressive breast cancers are characterized by increased expression of HBP enzymes and elevated metabolic flux, which simultaneously drive HA overproduction and hyper-*O*-GlcNAcylation. Rather than competing, these glycosylation pathways act in concert to reinforce pro-tumorigenic signaling networks and promote cancer progression [25]. Supporting this broader concept, Muniz de Queiroz et al. (2019) demonstrated that enhanced HBP-driven glycosylation — including increased *O*-GlcNAcylation as well as global *N*- and *O*-glycosylation — differentially modulates distinct molecular pathways while converging functionally to enhance cell motility and migration in melanoma cells [26]. These observations highlight that multiple glycosylation modalities, although mechanistically distinct, are coordinately regulated through UDP-GlcNAc metabolism to influence cellular behavior and disease progression.

## 3 Crosstalk between glycosylation pathways in human diseases

Building on this concept, we next focus on the crosstalk between glycosylation pathways in human diseases, highlighting how perturbations in one pathway can propagate to others and collectively reshape cellular functions. Special focus will be placed on the post-translational regulation of glycosylation enzymes and related proteins by *O*-GlcNAcylation, providing an additional, integrative layer of control over the glycosylation network (Figure 3). As a dynamic and reversible PTM, *O*-GlcNAcylation fine-tunes protein function by modulating protein–protein interactions, stability, subcellular localization, and enzymatic or transcriptional activity. Beyond the regulation of individual targets, aberrant *O*-GlcNAcylation is closely associated with the onset and progression of numerous diseases [27] and has emerged as a hallmark of mammalian cell pathophysiological activation [28,29]. In this context, elevated *O*-GlcNAcylation couples Warburg-like metabolic rewiring to coordinated transcriptional and signaling reprogramming, thereby enabling the acquisition of new cellular functionalities. Although increasing evidence supports a central role for *O*-GlcNAcylation in regulating glycosylation-related protein expression, activity, and intracellular trafficking [10], its contribution to the coordinated regulation of multiple glycosylation pathways in pathophysiological settings remains only partially explored. Here, we illustrate this crosstalk across a range of human diseases — including metabolic disorders, cardiovascular and neurological diseases, CDGs and cancers — highlighting how concerted or dysregulated glycosylation programs shape disease trajectories (Figure 3; Table 1).

a. Metabolic Disorders

**Table 1:** Crosstalk between glycosylation pathways in human diseases.

Diseases	Glycosylation mechanism	Functional consequence	Pathological outcome	References
**Metabolic diseases**	*O*-GlcNAcylation stabilizes SLC35A3, enhancing UDP-GlcNAc import into the Golgi and supporting GnT-IV-dependent *N*-glycan branching	Increased complex *N*-glycosylation of membrane proteins (e.g., glucose transporters), enhancing protein stability and cell surface residency	Dysregulation of the OGT-SLC35A3-GnT-IV axis may impair glucose transporters function, contributing to defective glucose sensing, insulin resistance and hyperglycemia in T2D	[30,[35], [36], [37]]
	OGT controls *O*- and *N*-glycosylation-related gene expression in hepatocytes	Maintenance of proper glycoprotein folding/maturation and ECM homeostasis	Hepatocyte-specific OGT loss induces *ER*/oxidative stress, DNA damage, inflammation, and progressive fibrosis, likely in part through impaired complex glycosylation	[43]
	*O*-GlcNAcylation stabilizes GS in a catalytically restrained state in adipocytes	Impaired glycogen synthesis essential for carbohydrate storage and cellular energy	Under nutrient excess, *O*-GlcNAc accumulation contributes to impaired glycogen storage, altered energy homeostasis, insulin resistance and β-cell dysfunction	[48,49]
	OGT-mediated *O*-GlcNAcylation stabilizes KANSL3 and supports NSL complex histone acetyltransferase activity	NSL directs transcriptional program essential for maintenance of hepatocyte identity and expression of liver detoxification genes (e.g., *UGT2A3*)	Impaired *O*-GlcNAcylation may disrupt NSL-dependent chromatin regulation, potentially contributing to loss of hepatic identity, biliary hyperplasia, and fibrosis	[[60], [61], [62]]
	Salivary α-amylase activity modulates starch digestion and postprandial glucose influx, and may indirectly regulate UDP-GlcNAc availability and *O*-GlcNAc signaling in adipocytes or muscle cells	*O*-GlcNAcylation modulates IRS/PI3K signaling	Low salivary α-amylase (*AMY1*) copy-number is associated with reduced insulin sensitivity and increased risk of T2D, possibly reflecting attenuation of insulin signaling by *O*-GlcNAc	[51,64]
	During fasting, NAGLU mediates lysosomal HS degradation and stabilizes OGT in skeletal muscle	OGT-mediated *O*-GlcNAcylation of PPARγ enhances its activity and induces glycosidase expression (e.g. *MAN2B1* and *HEXA*)	Chronic or excessive *O*-GlcNAcylation leads to β-dystroglycan deglycosylation, insulin receptor loss, and metabolic dysfunction	[53]
**Cardiovascular diseases**	*O*-GlcNAcylation stabilizes HAS2 (e.g., at Ser^221^), modulates its intracellular trafficking, enhances its expression *via O*-GlcNAc-dependent transcriptional regulation (YY1, SP1, NF-κB/p65 *via HAS2-AS1*), and may also coordinate UDP-sugar supply (e.g., UGP) for HA synthesis	Increased HA synthesis and pericellular matrix remodeling	Excessive *O*-GlcNAcylation drives pathological HA accumulation, contributing to vascular remodeling, inflammation, and cancer progression	[15,[72], [73], [74], [75], [76],^78^]
	*O*-GlcNAcylation stabilizes SIRT3, limiting HYAL2 activation and HA degradation	Maintenance of endothelial glycocalyx homeostasis under physiological shear stress	Loss of SIRT3 *O*-GlcNAcylation under disturbed flow promotes glycocalyx degradation and supports vascular inflammation	[79]
**Neurodegenerative diseases**	OGT-TET3 complex promotes *O*-GlcNAc-dependent chromatin remodeling at the GnT-IX promoter, facilitating NeuroD1 recruitment and transcriptional activation in Neuro-2A neural cells	Induction of GnT-IX expression for brain-specific *N*-glycan branching	Disruption of *O*-GlcNAc cycling may impair brain *N*-glycosylation, altering synaptic protein trafficking (e.g., SV2), compromising synaptic function, and promoting protein misfolding and aggregation (Aβ, tau, α-synuclein), thereby contributing to neurodegeneration	[83,84,92]
	In the prefrontal cortex, OGT regulates HEXA, a lysosomal enzyme essential for ganglioside turnover	HEXA-mediated maintenance of ganglioside metabolism and myelination processes in the brain	Impaired HEXA regulation may contribute to myelination defects and brain abnormalities observed in OGT-ID disease	[94,95]
	*O*-GlcNAc signaling regulates key glycogen-metabolizing enzymes (PYGM, GYG1, GYS1) in medial prefrontal cortex astrocytes	Coupling of glycosylation dynamics to glycogen metabolism and astrocyte-mediated energy support	OGT deficiency disrupts astrocytic glycogen metabolism and energy homeostasis, impairing the neuronal environment and contributing to neuropsychiatric disorders	[96]
**CDGs**	PMM2 deficiency (PMM2-CDG) causes *N*-glycosylation defects, with concomitant UDP-HexNAc accumulation and increased *O*-GlcNAcylation	*O*-GlcNAcylation stabilizes NgBR and enhances DPAGT1 activity, partially compensating early *N*-glycosylation steps	*O*-GlcNAc-mediated enhancement of the *N*-glycosylation machinery exerts a protective effect, which is associated with improved cartilage phenotype and reduced disease severity in PMM2-CDG	[106]
**Cancers**	*O*-GlcNAc marks co-localize with PRC2-associated chromatin repression and regulate glycosylation gene transcription (e.g., *ST3GAL1*) in colon cancer stem cells	Regulation of glycan remodeling programs that can control mucin-type *O*-glycan sialylation, a feature observed in cancer cells	Epigenetic *O*-GlcNAc regulation may contribute to cancer stem-like maintenance through altered glycosylation programs	[145]
	*O*-GlcNAc-modified chromatin-associated transcription factors regulate glycosylation genes (e.g., *MAN2C1*) in breast cancer cells	*MAN2C1* expression may be involved in adaptation to genotoxic stress	Potential OGT-MAN2C1 axis contribution to survival and stress tolerance in differentiated cancer cells under genotoxic stress	[146]
	OGT regulates expression of glycosidases and glycosyltransferases (*MANBA, ST8SIA6, GALNT8, GCNT3*)	OGT inhibition-associated remodeling of global cell-surface glycosylation (e.g. increased α2,3-sialylation, β1,6-GlcNAc branching of *N*-glycans, decreased GSL sialylation), and concomitant increased in E-cadherin expression, with altered glycosylation (reduced β1,6-GlcNAc branching and core fucosylation of *N*-glycans) and enhanced localization at cell-cell junctions	Contribution of OGT to EMT-associated phenotypes, potentially through reduced expression and increased β1,6-GlcNAc branching on E-cadherin, features commonly associated with impaired cell–cell adhesion and tumor progression	[122]
	Post-translational regulation, including *O*-GlcNAcylation, may modulate B4GALNT2 stability, trafficking or enzymatic activity in colon cancer cells	B4GALNT2 activity controls the balance between Sda antigen and sLeˣ tumor-associated glycan levels	Reduced B4GALNT2 activity is associated with a shift toward an sLeˣ-enriched glycome linked to pro-metastatic features; *O*-GlcNAcylation may contribute to the regulation of this process	[147]
	Glucagon- and hypoxia-induced *O*-GlcNAcylation of PYGL at Ser^430^ promotes phosphorylation at Ser^15^, enhancing enzymatic activity in colon cancer cells	Integration of nutrient sensing with glycogen metabolism and cellular metabolic stress responses	Under hypoxic and glucagon-induced metabolic stress conditions, *O*-GlcNAc-dependent regulation of PYGL activity promotes glycogen utilization, thereby supporting tumor metabolic adaptation	[127]
	*O*-GlcNAcylation stabilizes C1GalT1 via strengthened interaction with Cosmc, preventing proteasomal degradation in bladder cancer cells	Increased C1GalT1 stability promotes T-antigen synthesis on glycolysis-related proteins	*O*-GlcNAcylation-dependent stabilization of C1GalT1 enhances aberrant mucin-type *O*-glycans and supports glycolysis-associated metabolic reprogramming in tumor cells	[130]
	*O*-GlcNAcylation activates Akt/ERK signaling, which subsequently reduces FoxO3 stability and leads to decreased *MAN1A1* expression in cholangiocarcinoma cells	*O*-GlcNAc-dependent-downregulation of *MAN1A1* expression results in increased high-mannose *N*-glycans	Elevated *O*-GlcNAcylation is associated with increased high-mannose *N*-glycan presentation at the cell surface, which may contribute to enhanced metastatic potential in cancer	[148]
	PNG1 stabilizes OGT and together they regulate Nrf2-dependent stress signaling in intestinal stem cells and undifferentiated progenitors cells	Integration of glycosylation and stress-response pathways, coordinating cellular proliferation and survival programs	The PNG1-OGT-Nrf2 axis may contribute to metabolic stress adaptation in stem cell-derived diseases such as cancer	[151]

This table summarizes how *O*-GlcNAcylation functionally interacts with other glycosylation pathways — including, *N*- and *O*-glycosylation, glycosphingolipids (GSLs), and glycosaminoglycans (GAGs) — and related metabolic processes (e.g., glycogen metabolism and nucleotide sugar availability) across human diseases, including metabolic, cardiovascular, and neurodegenerative disorders, congenital disorders of glycosylation (CDGs), and cancers. For each condition, the underlying glycosylation-related mechanism, its functional consequences, and the associated pathological outcomes are presented. Together, these observations highlight a bidirectional crosstalk whereby glycosylation and metabolism coordinately shape cellular function and disease-associated phenotypes.

Aβ, amyloid beta; AMY1, Alpha-amylase 1; B4GALNT2, Beta-1,4-N-acetylgalactosaminyltransferase 2; C1GalT1, Core 1 β1,3-galactosyltransferase; CD44, Cluster of differentiation 44; Cosmc, Core 1 β3-Gal-T-specific molecular chaperone; DPAGT1, Dolichyl-phosphate N-acetylglucosaminephosphotransferase 1; ECM, extracellular matrix; EMT, epithelial-to-mesenchymal transition; *ER*, *endoplasmic reticulum*; ERK, Extracellular signal-regulated kinase; FoxO3, Forkhead box O3; GALNT8, Polypeptide N-acetylgalactosaminyltransferase 8; GCNT3, Glucosaminyl (N-acetyl) transferase 3; GnT, N-acetylglucosaminyltransferase; GS, Glycogen synthase; HA, hyaluronan; HAS, Hyaluronan synthase; HEXA, Hexosaminidase A; HYAL, Hyaluronidase; IRS, Insulin receptor substrate; KANSL3, Lysine acetyltransferase 8 regulatory nonspecific lethal complex subunit 3; MAN1A1, Mannosidase alpha class 1A member 1; MAN2B1, Mannosidase alpha class 2B member 1; MAN2C1, Mannosidase alpha class 2C member 1; MANBA, Mannosidase beta; NAGLU, α-N-acetylglucosaminidase; NF-κB, Nuclear factor kappa B; NeuroD1, Neurogenic differentiation 1; NgBR, Nogo-B receptor; Nrf2, Nuclear factor erythroid 2–related factor 2; NSL, Non-specific lethal complex; *O*-GlcNAc, *O*-linked N-acetylglucosamine; OGT, *O*-GlcNAc transferase; PI3K, Phosphoinositide 3-kinase; PMM2, Phosphomannomutase 2; PNG1, Peptide:*N*-glycanase 1; PPARγ, Peroxisome proliferator-activated receptor gamma; PRC2, Polycomb repressive complex 2; PYGL, Glycogen phosphorylase, liver form; SIRT3, Sirtuin 3; SLC35A3, Solute carrier family 35 member A3; SP1, Specificity protein 1; ST3GAL1, ST3 beta-galactoside alpha-2,3-sialyltransferase 1; ST8SIA6, ST8 alpha-*N*-acetyl-neuraminide alpha-2,8-sialyltransferase 6; SV2, Synaptic vesicle protein 2; TET3, Tet methylcytosine dioxygenase 3; UGP, UDP-glucose pyrophosphorylase; UGT2A3, UDP-glucuronosyltransferase family 2 member A3; UDP-GlcNAc, uridine diphosphate N-acetylglucosamine; UDP-HexNAc, uridine diphosphate N-acetylhexosamine; YY1, Yin yang 1.

Under physiological conditions, glycosylation acts as a nutrient-sensitive regulatory system that aligns cellular function with metabolic status. For instance, nutrient-dependent *N*-glycan branching on membrane receptors and solute transporters modulates their surface residency and signaling capacity, thereby fine-tuning insulin sensitivity and glucose homeostasis [30]. In parallel, dynamic *O*-GlcNAcylation of TFs and metabolic enzymes integrates nutrient availability with transcriptional programs and metabolic fluxes controlling glycolysis, lipogenesis, gluconeogenesis and glycogen synthesis [31]. Together, these complementary layers of regulation confer metabolic flexibility, allowing tissues to adapt to feeding–fasting cycles while maintaining functional integrity. However, this finely balanced system is challenged by modern dietary habits. Over the past five decades, global sugar consumption has tripled, particularly in emerging economies, paralleling the rising prevalence of metabolic disorders [32]. Chronic consumption of a Western-style diet, rich in sugars and saturated fats, perturbs glucose homeostasis and promotes the development of obesity, insulin resistance, T2D, and metabolic-associated fatty liver disease (MAFLD) [33,34]. These metabolic disturbances are more than a consequence of excess calories; they arise from profound shifts in cellular nutrient sensing, UDP-GlcNAc abundance and availability, and glycosylation pathways — including *N*-glycan branching, *O*-GlcNAcylation, and carbohydrate storage — collectively reshaping hepatic metabolism, hormone responsiveness, and energy homeostasis.

Elevated flux through the HBP increases UDP-GlcNAc levels, simultaneously affecting multiple glycosylation-dependent processes [17]. Aberrant *N*-glycan branching on nutrient transporters, such as Glucose transporter 4 (GLUT4) in adipose tissue and skeletal muscle, impairs membrane translocation and glucose uptake, fostering insulin resistance [35], while defective *N*-glycosylation of β-cell GLUT2 impairs insulin secretion [36]. In this context, *O*-GlcNAcylation functions as a critical integrator, modulating the expression, activity, and trafficking of glycosylation enzymes. For instance, through the OGT-SLC35A3-N-acetylglucosaminyltransferase IV (GnT-IV also referred as Alpha-1,3-mannosyl-glycoprotein 4-beta-N-acetylglucosaminyltransferase, MGAT4) axis, *O*-GlcNAcylation stabilizes the UDP-GlcNAc transporter SLC35A3 and promotes the biosynthesis of complex branched *N*-glycans. This regulatory node can ensure the stability and surface residency of GLUT2, supporting insulin secretion [37]. Dysregulation of this axis contributes to impaired glucose sensing, hyperglycemia, and the onset of T2D [38] (Table 1). This functional interplay between the HBP, *O*-GlcNAcylation, and *N*-glycan branching is further exemplified in hepatocytes. Li et al. (2025) demonstrated that glucose exposure simultaneously activates the HBP and the *de novo* lipogenesis (DNL) program, with intact HBP being essential for glucose-induced DNL activation and triglyceride (TG) accumulation. While HBP activation elevates both protein *O*-GlcNAcylation and *N*-linked glycosylation, flux through the *N*-glycosylation pathway predominates in driving lipogenesis [39]. This effect may involve the regulation of Sterol regulatory element-binding protein (SREBP)/SREBP cleavage-activating protein (SCAP) trafficking from the *ER* to the Golgi [40], a critical step for activation of SREBP and subsequent transcriptional control of DNL. Oral GlcNAc supplementation, which increases UDP-GlcNAc availability, promotes extensive *N*-glycan branching on hepatic cell surface glycoproteins, stabilizing key receptors and transporters [13,41], and enhancing nutrient uptake, lipid storage, and glycogen accumulation. While such adaptations can support energy homeostasis under physiological conditions, chronic or excessive *N*-glycan branching can lead to ectopic lipid deposition and hepatic steatosis, and disrupt the normal hepatic response to fasting, highlighting the central role of UDP-GlcNAc-driven *N*-glycan remodeling in hepatic metabolic control [42]. Consistently, hepatocyte-specific deletion of OGT (OGT^LKO^) disrupts this integrated network, broadly altering the expression of genes involved in *O*- and *N*-glycan-associated glycosylation, glycoprotein maturation, and ECM organization. The resulting loss of *O*-GlcNAc homeostasis triggers profound cellular stress responses, including oxidative and *ER* stresses, DNA damage, inflammation, and progressive fibrosis in the liver, underscoring the essential role of *O*-GlcNAcylation in coordinating metabolic fluxes, glycoprotein processing, and cellular homeostasis [43] (Table 1).

Beyond *N*- and *O*-glycan remodeling, carbohydrate storage, particularly glycogen, constitutes another glycosylation-dependent axis that is tightly intertwined with metabolic homeostasis. Deregulation of glycogen metabolism disrupts cellular energy balance and nutrient sensing compromising glucose homeostasis, pancreatic β-cell function, and insulin signaling [44]. Glycogen is not a mere energy reservoir; it functions as a dynamic metabolic buffer. Glucose uptake and phosphorylation generate glucose-6-phosphate (Glc-6-P), a pivotal metabolite partitioned between glycolysis, glycogen synthesis, and the HBP, which diverts a small but critical fraction toward UDP-GlcNAc production. Through this mechanism, glycogen supplies substrates that sustain both *N*-glycosylation [45] and *O*-GlcNAcylation [46] under nutrient-limited conditions. Conversely, *O*-GlcNAcylation modulates glycogen metabolism in a nutrient-sensitive manner, regulating enzyme function. Glycogen synthase (GS), the rate-limiting enzyme in glycogen synthesis, has been identified as an *O*-GlcNAc–modified protein [[47], [48], [49], [50]]. Parker and colleagues’ seminal studies showed that, in adipocytes and murine fat pad extracts, *O*-GlcNAc modification stabilizes GS in a Glc-6-P-dependent but restrained state, making dephosphorylation by Protein phosphatase 1 (PP1) insufficient for full enzymatic activation. This mechanism provides a direct link between nutrient excess, *O*-GlcNAc accumulation, and impaired glycogen synthesis in insulin-resistant and diabetic states [48,49]. The detection of *O*-GlcNAcylated GS in skeletal muscle proteomes [50], further supports the relevance of this regulatory axis in insulin-sensitive tissues central to postprandial glucose disposal. Together, these findings support a model in which glycogen metabolism and *O*-GlcNAcylation are engaged in a reciprocal regulatory loop, coupling carbohydrate storage to nutrient sensing and contributing to the dysregulation of glucose homeostasis observed in metabolic diseases (Table 1).

Key signaling nodes, including Phosphoinositide 3-kinase (PI3K)/AKT [51], as well as TFs such as Forkhead box protein O1 (FoxO1), Peroxisome proliferator-activated receptor gamma (PPAR-γ) coactivator 1-alpha (PGC1α), SREBP, and Carbohydrate-responsive element-binding protein (ChREBP) [52], are dynamically *O*-GlcNAcylated, modulating their activity, subcellular localization, and downstream gene expression [53]. Importantly, lipogenic enzymes themselves, including Fatty acid synthase (FAS) [[54], [55], [56], [57], [58]] and Acetyl-CoA carboxylase (ACC) [59], are also *O*-GlcNAcylated, providing a direct post-translational mechanism to fine-tune lipid synthesis. Through these modifications, *O*-GlcNAcylation coordinates metabolic programs that regulate lipogenesis and gluconeogenesis, ensuring integrated control of lipid and glucose metabolisms. Chronic perturbations of these modifications destabilize metabolic homeostasis and predispose to insulin resistance and metabolic diseases [51,53].

These regulatory effects extend to glycoprotein turnover, receptor homeostasis, and epigenetic control of hepatocyte identity. For instance, by stabilizing the Lysine acetyltransferase 8 regulatory nonspecific lethal complex subunit 3 (KANSL3) [60,61], *O*-GlcNAcylation could support the Non-Specific Lethal (NSL) complex–mediated histone acetylation and the expression of hepatocyte-specific genes such as UDP-glucuronosyltransferase family 2 member A3 (UGT2A3), which is essential for liver detoxification and metabolic homeostasis [62]. Perturbation of this regulatory axis — whether through insufficient *O*-GlcNAcylation [63] or loss of KANSL3 function [62] — compromises chromatin integrity and hepatic identity, contributing to pathologies, notably biliary hyperplasia and hepatic fibrosis. Upstream factors — including salivary α-amylase activity which is influenced by copy number polymorphisms — modulate starch digestion and the postprandial glucose influx into the HBP, thereby impacting the availability of UDP-GlcNAc. These shifts directly affect *O*-GlcNAc-mediated modulation of Insulin receptor substrate (IRS)/PI3K signaling, creating a mechanistic link between digestive efficiency, nutrient sensing, and insulin action [51,64]. Notably, variation in α-amylase gene copy number correlates with age-related declines in insulin sensitivity, implicating this upstream control point in the development of metabolic disorders such as T2D [64]. Conversely, chronic or excessive *O*-GlcNAcylation can perturb glycoprotein turnover and receptor homeostasis. For instance, lysosomal degradation of HS by α-N-acetylglucosaminidase (NAGLU) stabilizes OGT and promotes *O*-GlcNAcylation of PPAR-γ. This in turn induces glycosidase expression, leading to β-dystroglycan deglycosylation and insulin receptor loss [53]. Together, these findings reveal that *O*-GlcNAcylation does not merely relay metabolic information but actively restructures glycosylation-dependent signaling and transcriptional landscapes, thereby influencing the stability and plasticity of metabolic states (Table 1).

In summary, glycosylation acts as a metabolic rheostat, balancing adaptive responses to nutrient availability. Chronic disruption of this system, driven by modern diets and lifestyle, underlies the pathogenesis of obesity, T2D, and fatty liver disease, highlighting glycosylation pathways as potential therapeutic targets for metabolic disorders.

b. Cardiovascular Diseases

Protein glycosylation — including *N*- and *O*-linked glycans as well as intracellular *O*-GlcNAcylation — has emerged as a fundamental regulatory layer of cardiac and vascular homeostasis. By dynamically integrating metabolic cues with structural and signaling networks, glycosylation fine-tunes intercellular communication and adaptive responses in the cardiovascular system. In pathological contexts, dysregulation of glycosylation pathways contributes to altered cardiac structure, metabolic inflexibility, and vascular inflammation, thereby driving the initiation and progression of heart failure, ischemic injury, and atherosclerosis [65]. Within this framework, *N*-glycosylation critically controls the maturation, trafficking, and activity of cell-surface receptors and transporters — such as adhesion molecules, growth factor receptors, and ion channels — as well as secreted glycoproteins, including immunoglobulins. Through these mechanisms, *N*-linked glycans shape coordinated signaling between cardiomyocytes, endothelial cells, and immune cells, whereas aberrant *N*-glycosylation disrupts receptor-ligand interactions and promotes pathological remodeling and vascular inflammation [66,67]. Mucin-type *O*-glycosylation further contributes to cardiovascular integrity by regulating protein stability and ECM organization. Dysregulated GALNT expression results in structural cardiac defects and valvular abnormalities. In parallel, changes in the *O*-glycosylation of circulating lipoproteins, notably high-density lipoproteins (HDL), impair cholesterol handling and favor atherosclerotic cardiovascular disease [67]. Beyond protein-centered modifications, HA introduces a distinct and highly plastic layer of extracellular regulation. Through rapid synthesis and molecular weight-dependent signaling, HA dynamically remodels the vascular microenvironment, shifting from a homeostatic component of the endothelial glycocalyx to a proinflammatory matrix during injury and atherosclerosis [68]. At the intracellular level, protein *O*-GlcNAcylation exerts a highly context- and time-dependent influence on cardiac physiology by modulating the activity of TFs, kinases, and metabolic enzymes. Transient increases in *O*-GlcNAcylation constitute an adaptive stress response that confers cardioprotection during ischemia-reperfusion by preserving mitochondrial function, limiting oxidative damage, and reducing cardiomyocyte death. In contrast, sustained or excessive *O*-GlcNAcylation, as observed in diabetes or chronic metabolic stress, drives maladaptive remodelling, electrical instability, impaired contractility, and ultimately heart failure [65,69]. In conclusion, this glycosylation represents a critical and dual-faced regulator of cardiovascular health, offering both protective and pathological outcomes. A deeper understanding of its mechanisms holds significant promise for the development of innovative therapeutic strategies aimed at preventing and treating cardiovascular diseases.

Cellular availability of UDP-GlcNAc has emerged as a central determinant of GAG biosynthesis [15], positioning nutrient flux as a direct regulator of ECM composition. Within this framework, protein *O*-GlcNAcylation acts as a key integrative signal that translates intracellular metabolic status into HA production and matrix remodeling in cardiovascular tissues. Complementing this regulation, *N*-glycosylation shapes not only the folding, localization, and enzymatic activity of hyaluronan synthases (HAS) [15] and hyaluronidases (HYAL) [70], but also the function of HA receptors including Cluster of differentiation 44 (CD44) [71]. By modulating receptor conformation and accessibility of HA-binding sites, *N*-glycans fine-tune HA-CD44 interactions, balancing high- and low-affinity engagements and thereby influencing HA-dependent signaling, endothelial adhesion, and vascular inflammation. Together, these modifications integrate structural and metabolic cues to orchestrate HA-mediated matrix dynamics in health and disease.

Beyond simply reflecting substrate abundance, *O*-GlcNAcylation exerts multi-level control over HA synthesis by coordinating enzyme stability [72], gene regulation [73,74], and intracellular trafficking [75,76]. At the post-translational level, *O*-GlcNAcylation directly modulates the activity and lifespan of HAS. In vascular smooth muscle cells, modification of HAS2 at Ser^221^ markedly enhances enzyme stability [15], thereby potentially enhancing the pericellular matrix remodeling in pathological settings such as vascular disease and cancer. This direct control is complemented by transcriptional and epigenetic mechanisms that sustain HA synthesis under conditions of prolonged metabolic stress. Increased flux through the HBP promotes *O*-GlcNAcylation of TFs, including Yin yang 1 (YY1) and Specificity protein 1 (SP1), favoring *HAS2* expression, while hyperglycemic conditions further engage a transcriptional-epigenetic relay involving Nuclear factor kappa B (NF-κB)-dependent induction of the long non-coding HAS2 antisense RNA 1 (*HAS2-AS1*). Acting in *cis*, *HAS2-AS1* remodels chromatin accessibility at the *HAS2* locus, enabling durable transcriptional activation [74]. Metabolic regulation of HA synthesis is further reinforced upstream at the level of nucleotide sugar metabolism. *O*-GlcNAcylation of UDP-glucose pyrophosphorylase (UGP) [77] provides a potential mechanism to synchronize glucose utilization with the generation of HA precursors, thereby aligning glycogen metabolism with GAG biosynthesis [78]. Nutrient-dependent control of HAS transit through the Golgi apparatus, residence at the plasma membrane and recycling toward degradative compartments, determines enzyme availability at the cell surface [75,76]. In particular, HAS3 behaves as a metabolically sensitive isoform whose retention at the plasma membrane is favored by elevated UDP-sugar levels, whereas nutrient depletion promotes internalization and loss of activity [75]. Through reciprocal interplay with phosphorylation at key regulatory residues, *O*-GlcNAcylation fine-tunes HAS2 stability and trafficking, thereby coupling intracellular signaling states to ECM remodeling in a context-dependent manner [76]. *O*-GlcNAc–dependent stabilization of signaling complexes, notably those involving the mitochondrial deacetylase Sirtuin 3 (SIRT3), preserves glycocalyx homeostasis and restrains HA degradation under physiological shear stress. Conversely, loss of SIRT3 *O*-GlcNAcylation under disturbed flow conditions favors pro-degradative pathways, accelerating glycocalyx injury and promoting vascular inflammation [79].

Together, these converging mechanisms position *O*-GlcNAcylation as a central coordinator of HA biology, integrating nutrient sensing, transcriptional and epigenetic regulation, post-translational control, and membrane trafficking into a unified program of matrix plasticity. In the cardiovascular system, this regulatory axis is particularly relevant to endothelial glycocalyx integrity and vascular inflammation (Table 1).

c. Neurological Diseases

In the nervous system, glycosylation operates as an organizing principle that couples protein homeostasis, membrane dynamics, and metabolic signaling to neuronal function [[80], [81], [82]]. At the interface between the secretory pathway and synaptic membranes, *N*-glycosylation governs protein folding, stability, and trafficking, thereby ensuring the proper surface expression of receptors, ion channels, and synaptic vesicle components [80]. Consistent with this role, *N*-glycan modification of Synaptic vesicle protein 2 (SV2) is required for its proper trafficking and for synaptic vesicle formation [83]. Disruption of this regulatory layer compromises synaptic signaling fidelity and circuit stability, creating a permissive environment for neurodegeneration. In Alzheimer's disease (AD), abnormal *N*-glycosylation of the amyloid precursor protein (APP) promotes β-secretase (Beta-site APP cleaving enzyme 1, BACE1)-dependent cleavage, increasing the generation of amyloid-β (Aβ) peptides. These alterations also raise Aβ hydrophobicity and its tendency to aggregate, ultimately accelerating amyloid plaque formation. More broadly, defective *N*-glycosylation promotes protein misfolding and aggregation, contributing to the accumulation of amyloid-β, tau, and α-synuclein, pathological features shared across AD, Parkinson's disease (PD), and amyotrophic lateral sclerosis (ALS) [84]. Mucin-type *O*-glycosylation adds an additional dimension of control, shaping protein behavior at the cell surface and within the ECM by modulating solubility, secretion, and receptor activity [80]. Dysregulation of this pathway disrupts receptor-mediated signaling and extracellular interactions, thereby amplifying neurodegenerative processes [85]. Emerging evidence further indicates that mucin-type *O*-glycosylation is subject to sex-dependent remodeling, as observed in Huntington's disease (HD), suggesting that glycan patterns may reflect intrinsic, sex-specific modulators of disease progression [86]. At the intracellular level, *O*-GlcNAcylation integrates metabolic and stress signals into neuronal signaling networks by dynamically modulating transcriptional regulators, kinases, and cytoskeletal proteins. By preventing pathological hyperphosphorylation, it exerts neuroprotective mechanisms in neurons by maintaining appropriate protein trafficking, cleavage, and solubility [80]. When this regulatory layer is compromised, the balance between *O*-GlcNAcylation and phosphorylation shifts, favoring the aggregation of tau, α-synuclein, and Trans activation response (TAR) DNA-binding protein 43 (TDP-43) in AD, PD and ALS [[87], [88], [89]]. These intracellular alterations are further reinforced by glycan-dependent interactions involving proteoglycans, such as HS-mediated facilitation of amyloid-β and tau aggregation in AD [80,90], alongside the altered distribution of GPI-anchored proteins, leading to impaired prion protein processing in prion diseases [80,91].

Emerging evidence indicates that glycosylation pathways in the nervous system are intricately interconnected, with *O*-GlcNAcylation acting as a central integrator. The first demonstration that a GT can be directly regulated by OGT in concert with chromatin remodeling was provided for the brain-specific *N*-glycan branching enzyme GnT-IX (MGAT5B) [92]. Activation of the GnT-IX locus relies on the recruitment of OGT by Tet methylcytosine dioxygenase 3 (TET3), which facilitates binding of the neurogenesis TF Neurogenic differentiation 1 (NeuroD1) to the promoter, driving transcription. This study exemplifies how intracellular *O*-GlcNAcylation can orchestrate the expression of enzymes that sculpt the extracellular glycan landscape. Extending this concept, perturbation of *O*-GlcNAc cycling during neural differentiation of human embryonic stem cell (hESC) reveals its essential role in coordinating multiple glycosylation pathways. Inhibition of OGT alters the expression of several glycan-related proteins involved in *N*-glycan and *O*-glycan initiation and maturation, as wells as GAG biosynthesis, demonstrating that *O-*GlcNAcylation links intracellular signaling to the assembly of the complex glycan environment necessary for proper neuronal differentiation [93]. The importance of *O*-GlcNAcylation for brain development is further illustrated *in vivo* [94]. In the OGT^C921Y^ mouse model of OGT-linked intellectual disability (OGT-ID), missense variants in *OGT* induce behavioural deficits, including hyperactivity, impulsivity, and impaired associative learning, alongside profound brain structural abnormalities. Proteomic analyses of the prefrontal cortex reveal widespread perturbations, notably affecting Hexosaminidase subunit alpha (HEXA) [94], a lysosomal glycoprotein crucial for ganglioside metabolism and myelination [95]. This body of work highlights potential molecular mechanisms linking altered glycosylation to neuroanatomical and functional deficits. OGT deficiency-mediated disruption of *O*-GlcNAc signaling in medial prefrontal cortex astrocytes perturbs key glycogen metabolizing enzymes (Muscle glycogen phosphorylase (PYGM), Glycogenin 1 (GYG1) and Glycogen Synthase 1 (GYS1)), linking intracellular glycosylation dynamics to energy homeostasis and glial contributions to neuropsychiatric outcomes [96].

Together, these findings illustrate that glycosylation in the nervous system constitutes a deeply interconnected network in which *O*-GlcNAcylation coordinates intracellular signaling, enzymatic regulation, and extracellular glycan architecture. Disruption of this network impairs both neuronal and glial functions, providing a mechanistic framework that connects glycosylation cross-talk to neurodevelopmental and neurodegenerative pathologies (Table 1).

d. Congenital Disorders of Glycosylation (CDGs)

CDGs constitute a heterogeneous group of inherited metabolic diseases (IMD), most often transmitted in an autosomal recessive manner, that exemplify the systemic consequences of impaired glycan biosynthesis and remodeling [97,98]. Rather than affecting a single pathway or organ, CDGs arise from defects in core cellular processes such as nucleotidesugar metabolism, GT function, vesicular trafficking, and organelle homeostasis that operate across the cytoplasm and multiple compartments of the secretory and endomembrane systems. Since the initial description of CDG in twins by Jaak Jaeken in 1980 [99], the number of identified CDGs has increased exponentially, with currently 189 genes linked to approximately 200 distinct disorder phenotypes [98]. This pervasive disruption of glycosylation accounts for the striking clinical pleiotropy observed in CDGs. As discussed above, the central role of glycosylation in metabolic, cardiovascular, and neurological physiology is frequently mirrored in CDGs by complex multisystem phenotypes, which combine neurodevelopmental impairment, metabolic dysregulation, immune dysfunction, and progressive organ involvement. Although each individual CDG is rare, their collective burden is far from negligible, ranging from ultra-rare conditions reported in only a handful of patients to more prevalent forms such as Phosphomannomutase 2 (PMM2)-CDG, for which over a thousand cases have been diagnosed worldwide over the past four decades [97].

In addition to their genetic and clinical heterogeneity, CDGs have revealed a substantial degree of functional interdependence between glycosylation pathways that were long considered autonomous. Increasing evidence indicates that a primary defect in a single glycosylation route can propagate throughout the broader glycosylation network, reshaping cellular metabolism and eliciting compensatory or maladaptive responses in parallel glycan-modifying systems. CDGs caused by mutations in enzymes of the HBP — including GFAT1 [98,100], Glucosamine-phosphate N-acetyltransferase 1 (GNPNAT1) [98], PGM3 [101,102] or UDP-N-acetylglucosamine pyrophosphorylase 1 (UAP1) [103] — provide clear examples of “crossed glycosylation defects”. Because these enzymes directly control the intracellular pool of UDP-GlcNAc, their dysfunction impacts multiple glycosylation processes that depend on this nucleotidesugar. In congenital myasthenic syndromes (CMS) caused by *GFPT1* mutations, patient muscle samples display reduced global protein *O*-GlcNAcylation, whereas overall *N*-glycosylation appears largely maintained [100]. This apparent dissociation suggests that *N*-glycosylation defects may not manifest as a uniform reduction in total *N*-glycan abundance but rather as protein- or site-specific alterations. Indeed, impaired occupancy of *N*-glycosylation sites or selective hypo-glycosylation of key synaptic glycoproteins may be sufficient to disrupt neuromuscular transmission [104]. Consistent with this notion, Holland et al. recently demonstrated hypo-*O*-glycosylation of the Acetylcholine receptor delta (AChRδ) subunit in *GFPT1*-deficient models, directly linking specific altered glycoprotein to synaptic dysfunction [105]. In contrast, CDGs associated with *PGM3* mutations display a more global glycosylation phenotype. In these patients, reduced *PGM3* enzymatic activity leads to a marked decrease in UDP-GlcNAc level, accompanied by concomitant reductions in both *O*-linked and *N*-linked protein glycosylation in derived immune cells and in serum [101,102].

Importantly, defects in nucleotide-sugar biosynthetic pathways that primarily fuel secretory glycosylation can also indirectly reshape the UDP-GlcNAc pool and thereby influence derived glycosylation processes. This is exemplified by deficiencies in Mannose phosphate isomerase (MPI) and PMM2, which catalyze the key steps in the synthesis of GDP-Man, a key donor for *N*-linked glycosylation, as well as for GPI anchor biosynthesis and *O*-mannosylation, from fructose-6-phosphate (Fru-6-P). Similarly, PGM1 deficiency affects the production of UDP-glucose (UDP-Glc) and, by extension, UDP-galactose (UDP-Gal) production from glucose-6-phosphate (Glc-6-P), two essential nucleotide sugars required for *N*-glycan elongation and branching, *O*-glycosylation and glycolipid biosynthesis. Although these enzymes are not part of the HBP, they act upstream of the pivotal intermediates Glc-6-P and Fru-6-P that directly supply the HBP. Defects in these pathways therefore propagate through central carbon metabolism, altering substrate partitioning and rerouting glucose-derived flux toward the HBP. This metabolic rewiring profoundly reshapes nucleotide sugar homeostasis and positions UDP-GlcNAc as a sensitive integrator of secretory pathway dysfunction. A striking example is PMM2-CDG, the most common form of CDG, in which the enzymatic defect is associated with accumulation of UDP-HexNAc — likely reflecting increased UDP-GlcNAc — and markedly elevated protein *O*-GlcNAcylation. Elevated *O*-GlcNAcylation stabilizes the Nogo-B receptor (NgBR) and enhances the activity of Dolichyl-phosphate N-acetylglucosaminephosphotransferase 1 (DPAGT1), two key regulators of the early steps of *N*-linked glycosylation, thereby improving cartilage phenotypes [106]. These findings provide direct evidence that *O*-GlcNAcylation can feed back onto the *N*-glycosylation machinery acting as a protective mechanism and revealing an unexpected level of coordination between these pathways (Table 1). A similar metabolic logic appears to extend to other CDGs affecting nucleotidesugar interconversion. Elevated UDP-GlcNAc levels have been reported in PGM1 deficiency [107], whereas MPI deficiency is associated with reduced *N*-glycosylation accompanied by a pronounced increase in protein *O*-GlcNAcylation [108]. Collectively, these disorders point to a shared adaptive response in which impaired secretory glycosylation redirects metabolic flux toward the HBP, leading to intracellular accumulation of UDP-GlcNAc and increased protein *O*-GlcNAcylation, while complex glycosylation remains defective. This supports a unifying model in which nucleotide-sugar imbalances act not only as biochemical constraints but also as metabolic signals that reshape glycosylation across multiple regulatory levels. In this context, UDP-GlcNAc availability and *O*-GlcNAcylation emerge as key links between cellular metabolism and glycosylation homeostasis, potentially contributing to phenotypic variability and disease severity in CDGs.

This crosstalk extends beyond glycan synthesis to vesicular trafficking, a central process for glycoprotein maturation, compartmentalization, and distribution [109]. Core components of the Coat protein complex II (COPII) *ER* exit complex, which mediates *ER*-to-Golgi transport, are dynamically modified by *O*-GlcNAc in a nutrient-sensitive manner. *O*-GlcNAcylation of Sec23 [110], Sec24 [111] and Sec31 [112] modulates protein–protein interactions during outer COPII coat assembly and disassembly, alters vesicle formation kinetics, and influences trafficking through the early secretory pathway. The COPγ1 subunit of the COPI coat is also *O*-GlcNAcylated, but its role in Golgi-to-*ER* trafficking requires further investigation [113]. Similarly, *O*-GlcNAcylation of Golgi stacking proteins such as Golgi reassembly stacking protein of 55 kDa (GRASP55) affects Golgi organization and autophagy-related pathways. Under nutrient deprivation, loss of *O*-GlcNAcylation promotes the relocalization of GRASP55 from the Golgi to autophagosome-lysosome contact sites, where it acts as a membrane tether to facilitate vesicle fusion [114]. *O*-GlcNAcylation further shapes membrane trafficking at the level of vesicle fusion by modulating Soluble NSF attachment protein receptors (SNARE) complex assembly [109]. Dynamic *O*-GlcNAc cycling of Synaptosome associated protein 29 (SNAP-29) regulates autophagosome-lysosome fusion [115], while de-*O*-GlcNAcylation of SNAP-23 promotes the formation of the Vesicle-associated membrane protein 8 (VAMP8)-SNAP-23-Syntaxin 4 (STX4) SNARE complex, accelerating exosome release [116]. Moreover, OGT inhibition causes perinuclear accumulation of lysosomes and late endosomes with defective small GTPase Rab7 recruitment, indicating that OGT activity is required for endolysosomal system homeostasis [117]. In parallel, *O*-GlcNAcylation of cargo proteins such as αB-crystallin [118] and Heterogeneous nuclear ribonucleoproteins A2/B1 (hnRNPA2B1) [119] biases their secretion into extracellular space by exosome. Thus, *O*-GlcNAcylation integrates metabolism with the secretory pathway, coordinating COP dynamics, Golgi organization, vesicle trafficking, and exosome-mediated protein export.

OGT sits at the core of cellular glycosylation networks, and its disruption causes distinct forms of CDG (OGT-CDG) [120]. To date, seventeen pathogenic *OGT* variants have been identified, affecting both the tetratricopeptide repeat (TPR) domain, which mediates protein–protein interactions, and the catalytic domain responsible for the GlcNAc transfer. This distribution highlights that pathogenic mutations can impair either substrate recognition or enzymatic activity. Clinically, OGT-CDG (also referred to as OGT-ID) is characterized by a conserved core phenotype of intellectual disability and developmental delay, often affecting speech and language acquisition. Beyond these shared features, patients exhibit a heterogeneous array of secondary manifestations, including behavioural abnormalities such as autism spectrum disorder (ASD) and attention-deficit/hyperactivity disorder (ADHD), as wells as brain, facial and muscular abnormalities. Recent report further suggests that the impact of OGT deficiency may extend beyond the nervous system. Notably, the occurrence of hepatoblastoma in an infant with OGT-CDG indicates that impaired *O*-GlcNAcylation could contribute to broader liver dysfunction and may increase susceptibility to malignancy [121]. The variability in clinical presentation likely reflects hypo-glycosylation of specific OGT substrates, resulting either from reduced catalytic activity of variants within the enzyme's catalytic domain or from altered protein–protein interactions in the TPR domain, which indirectly disrupt substrate glycosylation [120]. The heterogeneity of OGT-CDG likely reflects widespread perturbations in the *O*-GlcNAc-modified proteome and its regulatory network. In line with this view, several studies have demonstrated that OGT interacts with and glycosylates multiple transcription regulators, some of which are themselves causally linked to ID phenotypes [120], thereby influencing gene expression programs involved in pluripotency, neurodevelopment, and synaptic function. Notable ID-associated OGT interactors include SPEN, a key mediator of X-chromosome inactivation; the nucleosome remodeler Bromodomain PHD finger transcription factor (BPTF); AT-hook DNA-binding motif-containing protein 1 (AHDC1), Additional sex combs like transcription regulator 2 (ASXL2), CREB-binding lysine acetyltransferase (CREBBP) and SET domain containing 1A, histone lysine methyltransferase (SETD1A), which coordinate epigenetic regulation through histone and DNA modifications; Nucleoporin 62 (NUP62) involved in nucleocytoplasmic transport; the RNA-processing factors Prolin-rich protein 12 (PRR12) and Zinc finger CCCH-type containing 14 (ZC3H14); and transcription factors such as Host cell factor 1 (HCFC1) [120]. Given the broad roles of glycosylation network dysregulation in neurodegenerative, metabolic, cardiovascular disorders, and cancer (as discussed throughout this review), it is plausible that OGT deficiency-driven transcriptional regulation may alter the expression of some GTs, GHs, and other proteins involved in glycosylation pathways, further contributing to the complex molecular and clinical manifestations of OGT-CDG. This concept is supported by transcriptomic analyses across multiple contexts of OGT loss or disrupted function, including neuronal differentiation [93], liver disease [43], and cancer [122], as wells as OGT-CDG patient-cells and disease models [123,124]. Complementing these findings, high-throughput mass spectrometry (HT-MS)-based proteomic data from the *O*-GlcNAcAtlas (https://oglcnac.org/atlas/) and the *O*-GlcNAc databases (www.oglcnac.mcw.edu) [125,126] reveal that numerous putative *O*-GlcNAc targets include enzymes and regulators of glycan biosynthesis and processing (Table 2). Although many of these proteins are membrane-associated or involved in the secretory pathway, the presence of cytoplasm-facing domains makes them accessible to OGT, providing a structural basis for direct regulation. Notably, beyond well-established OGT targets such as HAS2 [15,76], GS [[47], [48], [49], [50]], Glycogen phosphorylase (GP) [127], Ryanodine receptor 2 (RYR2) [128], and OGT and OGA themselves [129], this analysis also highlights a novel subset of glycosylation-related proteins with a high confidence of OGT-mediated-*O*-GlcNAcylation. These comprise cytosolic and nuclear factors such as Glycogen debranching enzyme (GDE, encoded by *AGL* gene), Neutral α-glucosidase C (GANC), Endo-β-N-acetylglucosaminidase (ENGase), Replication termination factor 2 (RTF2), alongside cytosolic domains of *ER* and Golgi-associated proteins including Dol-P-Glc:Glc(2)Man(9)GlcNAc(2)-PP-Dol alpha-1,2-glucosyltransferase A (ALG10), Copper-transporting ATPase 1 (ATP7A), Copper-transporting ATPase 2 (ATP7B), and Inositol 1,4,5-trisphosphate receptor type 2 (ITPR2) (Table 2). These proteins suggest an expanded *O*-GlcNAc-associated glycosylation network that extends beyond canonical metabolic regulators to include components of ion homeostasis, trafficking, and glycan processing pathways. This cytosolic paradigm does not readily explain reported *O*-GlcNAcylation events within luminal domains of secretory pathway enzymes. Intriguingly, OGT-dependent regulation of the stability of the mucin-type *O*-glycan enzyme Core 1 β1,3-galactosyltransferase 1 (C1GalT1) has been previously reported [130]. Notably, the putative *O*-GlcNAc sites Thr^229^ and Thr^233^, located within the luminal domain of C1GalT1 and implicated in the regulation of enzyme stability, were initially not experimentally demonstrated to be *O*-GlcNAcylated, but instead predicted using the YinOYang 1.2 server. Nevertheless, the reduced *O*-GlcNAc signal upon mutagenesis on these residues and following modulation of OGT levels, was interpreted as evidence consistent with OGT-dependent regulation. Given the canonical segregation of OGT within the cytosolic compartment and the luminal localization of C1GalT1, these findings raise an unresolved topological issue regarding how this modification is achieved. Although the underlying mechanism remains unclear, these observations suggest that *O*-GlcNAcylation could occur at an early stage of protein biogenesis, potentially prior to or during *ER* translocation [131]. Consistent with this view, *O*-GlcNAcylation has been shown to stabilize nascent polypeptides and protect them from premature ubiquitin-dependent proteasomal degradation, supporting a broader role in protein maturation and proteostasis [132].

**Table 2:** Putative *O*-GlcNAcylated glycosylation-related proteins.

	Gene symbol	UniProt Protein name	UniProt protein accession	UniProt subcellular location	Identified *O*-GlcNAc sites and topology localisation	References
GTs	**ALG10**	Dol-P-Glc:Glc(2)Man(9)GlcNAc(2)-PP-Dol alpha-1,2-glucosyltransferase A	Q5BKT4	*ER* membrane	T314 (cytoplasmic domain)	[216,217]
	**B3GLCT**	Beta-1,3-glucosyltransferase	Q6Y288	*ER* membrane	S125 (luminal domain)	[218]
	**B4GALT1**	Beta-1,4-galactosyltransferase 1	P15291	Golgi apparatus, Golgi stack membrane, cell membrane, cell surface, cell projection, filopodium, secreted	S112 (luminal domain)	[219]
	**EXTL3**	Exostosin-like 3	O43909	*ER* membrane, Golgi apparatus, cell membrane, nucleus	T594, T596 (luminal domain)	[219]
	**GALNT1**	Polypeptide N-acetylgalactosaminyltransferase 1	Q10472	Golgi apparatus, Golgi stack membrane, secreted	T554 (luminal domain)	[219]
	**GALNT4**	Polypeptide N-acetylgalactosaminyltransferase 4	Q8N4A0	Golgi apparatus membrane	T379 (luminal domain)	[220]
	**GALNT14**	Polypeptide N-acetylgalactosaminyltransferase 14	Q96FL9	Golgi apparatus membrane	T19 (transmembrane domain)	[220]
	**GALNT2**	Polypeptide N-acetylgalactosaminyltransferase 2	Q10471	Golgi apparatus, Golgi stack membrane, secreted	S59, S67, T70, S402 (luminal domain)	[221]
	**GALNT7**	N-acetylgalactosaminyltransferase 7	Q86SF2	Golgi apparatus membrane	T121 (luminal domain)	[221]
	**GLT8D1**	Glycosyltransferase 8 domain-containing protein 1	Q68CQ7	Golgi apparatus membrane	T278, S282 (luminal domain)	[220]
	**GYS1**	Glycogen [starch] synthase, muscle	P13807	Cytoplasm, cytosol, inclusion body, membrane	S698	[222]
	**HAS2**	Hyaluronan synthase 2	Q92819	Cell membrane, *ER* membrane, vesicle, Golgi apparatus membrane, lysosome	S180, S221 (cytoplasmic domain)	[15,76,223]
	**MGAT2**	Alpha-1,6-mannosyl-glycoprotein 2-beta-N-acetylglucosaminyltransferase	Q10469	Golgi apparatus membrane	S59 (luminal domain)	[224]
	**MGAT5B**	Alpha-1,6-mannosylglycoprotein 6-beta-N-acetylglucosaminyltransferase B	Q3V5L5	Golgi apparatus membrane	S129 (luminal domain)	[225]
	**OGT**	UDP-N-acetylglucosamine--peptide N-acetylglucosaminyltransferase 110 kDa subunit	O15294	Nucleus, cytoplasm, mitochondrion, membrane, cell membrane, mitochondrion membrane, cell projection	S10, T12, S18, T38, S52, S56, S391, T393, T449	[[226], [227], [228]]
	**POGLUT1**	Protein *O*-glucosyltransferase 1	Q8NBL1	*ER* lumen	S205	[218]
	**POGLUT2**	Protein *O*-glucosyltransferase 2	Q6UW63	*ER* membrane	S303	[218]
	**POGLUT3**	Protein *O*-glucosyltransferase 3	Q7Z4H8	*ER* lumen	T252, S265, S21	[218]
	**POMGNT2**	Protein *O*-linked-mannose beta-1,4-N-acetylglucosaminyltransferase 2	Q8NAT1	*ER* membrane	T43 (luminal domain)	[218]
	**POMT1**	Protein O-mannosyl-transferase 1	Q9Y6A1	*ER* membrane	S541 (luminal domain)	[218]
	**PYGL**	Glycogen phosphorylase, liver form	P06737	Cytoplasm, cytosol	S430	[127]
	**RTF2**	Replication termination factor 2	Q9BY42	Chromosome	S268, S280	[223]
	**ST3GAL4**	CMP-N-acetylneuraminate-beta-galactosamide-alpha-2,3-sialyltransferase 4	Q11206	Golgi apparatus, Golgi stack membrane, secreted	S132 (luminal domain)	[218]
	**ST6GALNAC1**	Alpha-N-acetylgalactosaminide alpha-2,6-sialyltransferase 1	Q9NSC7	Golgi apparatus membrane	T171, T175, S177, T364 (luminal domain)	[229]
	**ST6GALNAC4**	Alpha-N-acetyl-neuraminyl-2,3-beta-galactosyl-1,3-N-acetyl-galactosaminide alpha-2,6-sialyltransferase	Q9H4F1	Golgi apparatus membrane	S137 (luminal domain)	[218]
	**STT3A**	Dolichyl-diphosphooligosaccharide--protein glycosyltransferase subunit STT3A	P46977	*ER*, *ER* membrane	S124 (transmembrane domain), T539, T546, T550, S553 (luminal domain)	[216,219,220,[230], [231], [232], [233]]
	**STT3B**	Dolichyl-diphosphooligosaccharide--protein glycosyltransferase subunit STT3B	Q8TCJ2	*ER*, *ER* membrane	T619, T625, S629 (luminal domain)	[216,[230], [231], [232]]
	**UGGT1**	UDP-glucose:glycoprotein glucosyltransferase 1	Q9NYU2	*ER* lumen, *ER*-Golgi intermediate compartment	T266	[231]
	**UGT2A3**	UDP-glucuronosyltransferase 2A3	Q6UWM9	Membrane	T417, S420 (extracellular domain)	[223]
	**UGT8**	2-hydroxyacylsphingosine 1-beta-galactosyltransferase	Q16880	Membrane, *ER*	S79 (extracellular domain)	[216,232,234]
	**XXYLT1**	Xyloside xylosyltransferase 1	Q8NBI6	*ER* membrane	S67, S77 (luminal domain)	[221,224,225]
GHs	**AGL**	Glycogen debranching enzyme	P35573	Cytoplasm	T1130, T1474	[223]
	**ENGASE**	Cytosolic endo-beta-N-acetylglucosaminidase	Q8NFI3	Cytoplasm, cytosol	S397	[220]
	**FUCA1**	Tissue alpha-L-fucosidase	P04066	Lysosome	T383	[216]
	**GAA**	Lysosomal alpha-glucosidase	P10253	Lysosome, lysosome membrane	S144, T153, T884	[218,[235], [236], [237]]
	**GANAB**	Neutral alpha-glucosidase AB	Q14697	*ER*, Golgi apparatus, melanosome	T451	[238]
	**GANC**	Neutral alpha-glucosidase C	Q8TET4	*n.d. by* UniProt Cytoplasm, nucleoplasm [239]	*n.d.*	[223]
	**GBA1**	Lysosomal acid glucosylceramidase	P04062	Lysosome membrane	S187, S310	[216,231]
	**GUSB**	Beta-glucuronidase	P08236	Lysosome	T274	[216,231]
	**HPSE**	Heparanase	Q9Y251	Lysosome membrane, secreted, nucleus	S292, T294	[230]
	**MAN1B1**	Endoplasmic reticulum mannosyl-oligosaccharide 1,2-alpha-mannosidase	Q9UKM7	*ER* membrane	T230 (luminal domain)	[221]
	**MAN2A2**	Alpha-mannosidase 2x	P49641	Golgi apparatus membrane	S516 (luminal domain)	[220]
	**MAN2B1**	Lysosomal alpha-mannosidase	O00754	Lysosome lumen, secreted	S932	[216,218]
	**MOGS**	Mannosyl-oligosaccharide glucosidase	Q13724	*ER* membrane	T127 (luminal domain)	[230]
	**OGA**	Protein *O*-GlcNAcase	O60502	Nucleus, cytoplasm	S398, S399, S405, S410, T415	[216,219,220,223,231]
Other glycan-related proteins	**AGA**	N(4)-(beta-N-acetylglucosaminyl)-L-asparaginase	P20933	Lysosome	T40	[218]
	**ARSK**	Arylsulfatase K	Q6UWY0	Secreted, lysosome	S235	[219]
	**ATP2A2**	Sarcoplasmic/endoplasmic reticulum calcium ATPase 2	P16615	*ER* membrane, sarcoplasmic reticulum membrane	S829 (transmembrane domain)	[233]
	**ATP7A**	Copper-transporting ATPase 1	Q04656	Golgi apparatus, trans-Golgi network membrane, cell membrane, melanosome membrane, early endosome membrane, cell projection, axon, dendrite, polysynaptic density, cytoplasm, cytosol, *ER*	T49 (cytoplasmic domain)	[233]
	**ATP7B**	Copper-transporting ATPase 2	P35670	Golgi apparatus, trans-Golgi network membrane, late endosome, Golgi apparatus membrane, cytoplasm, mitochondrion	T788 (cytoplasmic domain)	[220]
	**CALR**	Calreticulin	P27797	*ER* lumen, cytoplasm, cytosol, secreted, extracellular space, extracellular matrix, cell surface, sarcoplasmic reticulum lumen, cytoplasmic vesicle, secretory vesicle, cortical granule, cytolytic granule	T325	[220]
	**CANX**	Calnexin	P27824	*ER* membrane, mitochondrion membrane, melanosome membrane	T59, T66, S74 (luminal domain)	[221,224,230,237,240]
	**CHST11**	Carbohydrate sulfotransferase 11	Q9NPF2	Golgi apparatus membrane	T225 (luminal domain)	[218]
	**CHST12**	Carbohydrate sulfotransferase 12	Q9NRB3	Golgi apparatus membrane	S96 (luminal domain)	[221]
	**CHST14**	Carbohydrate sulfotransferase 14	Q8NCH0	Golgi apparatus membrane	S252, T259 (luminal domain)	[221,224]
	**ERO1A**	ERO1-like protein alpha	Q96HE7	*ER* membrane, Golgi apparatus lumen, secreted, cell projection, dendrite	T282	[216]
	**HS6ST2**	Heparan-sulfate 6-O-sulfotransferase 2	Q96MM7	Membrane	S591 (luminal domain)	[216]
	**HSP90B1**	Endoplasmin	P14625	*ER* lumen, sarcoplasmic reticulum lumen, melanosome	S64, T219, T490, T604	[216,219,231,238,241]
	**HSPA5**	Endoplasmic reticulum chaperone BiP	P11021	*ER* lumen, melanosome, cytoplasm, cell surface	S637	[231,237]
	**HYOU1**	Hypoxia up-regulated protein 1	Q9Y4L1	*ER* lumen	S513, T517, S583, T589, T590, T598, T727, S832, T864, T871, S933	[216,217,219,221,230,231,237,238,242]
	**ITPR2**	Inositol 1,4,5-trisphosphate-gated calcium channel ITPR2	Q14571	*ER* membrane, cytoplasmic vesicle, secretory vesicle membrane	T1789 (cytoplasmic domain)	[220]
	**PDIA3**	Protein disulfide-isomerase A3	P30101	*ER*, *ER* lumen, melanosome	T120, S126, T485	[220,224]
	**PDIA4**	Protein disulfide-isomerase A4	P13667	*ER* lumen, melanosome	S135, T254, S371	[238]
	**POMK**	Protein *O*-mannose kinase	Q9H5K3	Endoplasmic reticulum membrane	S69 (luminal domain)	[218]
	**PPIB**	Peptidyl-prolyl *cis-trans* isomerase B	P23284	Virion, *ER* lumen, melanosome	S139	[243]
	**PRKCSH**	Glucosidase 2 subunit beta	P14314	*ER*	S282, T287, S293, T298, T469	[218,220,221,225,230,240,244,245]
	**RYR2**	Ryanodine receptor 2	Q92736	Sarcoplasmic reticulum membrane	T1468 (cytoplasmic domain)	[128,236]
	**UST**	Uronyl 2-sulfotransferase	Q9Y2C2	Golgi apparatus membrane	S136, S157 (luminal domain)	[218]

This table complies human GTs, GHs, and glycan-related proteins including nucleotide-sugar transporters and metal ion regulators, involved in glycosylation processes that have been reported as putative *O*-GlcNAc targets. Candidate glyco-enzymes were initially identified using the *Repository of Glyco-Enzyme Expression Constructs* (https://glycoenzymes.ccrc.uga.edu/) and [246,247], and were subsequently cross-referenced across two independent curated resources of HT-MS data, the *O-GlcNAc Database* (https://www.oglcnac.mcw.edu/) [125,126] and the *O*-GlcNAcAtlas database (https://oglcnac.org/atlas/) [248,249], to document evidence of *O*-GlcNAcylation, annotated modification sites, and associated literature. Subcellular localization was integrated from UniProt (https://www.uniprot.org/) and topology predictions derived from UniProt and Protter (https://protter.ethz.ch/start/), allowing assessment of intracellular compartmentalization and site accessibility. For proteins exhibiting luminal/extracellular localization of reported *O*-GlcNAc sites, the sequence context surrounding the identified modification sites was assessed for the presence of the EOGT consensus motif CXXG(Y/F)(T/S)GX_2_–_3_C. None of the reported *O*-GlcNAc sites were found within this consensus motif, suggesting that these positions are unlikely to represent canonical EOGT substrates. Alternatively, these observations may reflect either OGT-mediated *O*-GlcNAcylation occurring at early stages of protein biogenesis, or misassignment of HexNAc modifications due to the identical monoisotopic mass of GlcNAc and GalNAc (203.079 Da). While this multi-source integration increases confidence in site assignment, the proteins listed here should be considered candidate rather than definitively validated *O*-GlcNAc substrates. Underlined proteins denotes high-confidence candidates of OGT-associated *O*-GlcNAcylation, supported by nuclear or cytoplasmic localization and structurally accessible Ser/Thr residues, including cytosol-exposed domains of membrane proteins. Overall, this dataset is not exhaustive and remains subject to limitations inherent to large-scale proteomic approaches, underscoring the need for targeted biochemical and cellular validation to confirm direct *O*-GlcNAcylation, define *O*-GlcNAc-mediated enzyme and target *O*-GlcNAc sites, and assess functional consequence.

*ER, endoplasmic reticulum*; *n.d., non defined.*

An additional layer of complexity arises from the existence of an alternative *O*-GlcNAc transferase operating within the secretory pathway, which may contribute to *O*-GlcNAc site assignments within luminal or extracellular domains that are otherwise difficult to reconcile with canonical OGT activity. Epidermal growth factor-like domain-specific *O*-GlcNAc transferase (EOGT) is a luminal *ER* enzyme that modifies Ser/Thr residues within a subset of Epidermal growth factor (EGF) domains in secreted and transmembrane proteins. EOGT specifically targets residues located between the fifth and sixth conserved cysteines of EGF repeats, within the consensus motif CXXG(Y/F)(T/S)GX_2_–_3_C [133], thereby restricting its activity to proteins harboring compatible EGF-like domains. In contrast to OGT, which regulates cytoplasmic, nuclear, and mitochondrial proteins, EOGT directly modulates pericellular proteins function through extracellular *O*-GlcNAc modification, such as by potentiating Notch receptor-ligand binding [134,135]. The functional relevance of this modification in the extracellular matrix is underscored by studies in *Drosophila*, where loss of EOGT leads to defects in the apical ECM [136]. Importantly, the identification of an EOGT-CDG validates this pathway as a *bona fide* glycosylation system whose disruption leads to human disease. Patients with EOGT-CDG present developmental abnormalities consistent with impaired ECM [137]. These observations highlight the need for cautious interpretation of *O*-GlcNAc proteomic data: for instance, Thr^847^ *O*-GlcNAc on the extracellular EGF-like domains of Perlecan (Heparan sulfate proteoglycan 2, HSPG2) likely reflects EOGT-mediated glycosylation rather than canonical OGT activity [138]. The mammalian EOGT substrate repertoire remains incompletely defined. Beyond HSPG2, *O*-GlcNAc modifications within EGF repeats have been reported on several additional proteins not classically associated with glycosylation pathways, including Notch receptor 1 (NOTCH1), NOTCH2, Neuronal epidermal growth factor-like protein 1 (NELL1), Laminin subunit alpha-5 (LAMA5), Peptidase domain containing associated with muscle regeneration 1 (PAMR1), Aminoacyl tRNA synthetase complex-interacting multifunctional protein 1 (AIMP1), and Thrombospondin-1 (TSP-1) [133], suggesting a broader — yet still emerging — substrate landscape. For instance, the absence of the EOGT consensus motif in the sequence context of reported luminal or extracellular sites within putative *O*-GlcNAcylated glycosylation-related proteins (Table 2) suggests potential limitations in HT-MS-based site annotation, including false-positive assignments, and/or glycosylation events that are not consistent with canonical EOGT substrate specificity. Accordingly, the candidate *O*-GlcNAcylated glycosylation-related proteins listed in Table 2 require protein-specific experimental validation to confirm enzyme specificity, site occupancy, and functional relevance.

Taken together, these observations suggest that OGT deficiency perturbs glycosylation not merely by reducing global *O*-GlcNAcylation levels, but by destabilizing a finely tuned interaction network that coordinates metabolism, trafficking, and glycan synthesis. Disruption of this OGT-centered interactome is therefore likely to propagate across glycosylation pathways, compromise adaptive responses to metabolic stress, and contribute to the multisystem dysfunction characteristic of CDGs.

e. Cancers

A hallmark of cancer cells is the Warburg effect, a metabolic reprogramming that favors aerobic glycolysis even in the presence of sufficient oxygen [139,140]. Beyond supporting rapid ATP production, this metabolic shift fuels anabolic pathways by increasing glucose and glutamine uptake and metabolism, thereby sustaining the biosynthetic needs of rapid proliferation. A major consequence of this rewiring is the enhanced flux through the HBP, which elevates intracellular UDP-GlcNAc levels and directly couples oncogenic metabolism to extensive remodeling of the cellular glycome [141]. Accordingly, altered glycosylation has emerged as a hallmark of malignancy and a long-standing source of diagnostic and prognostic biomarkers. Increased branching of *N*-glycans, aberrant sialylation such as sialyl-Lewis antigen expression, truncation of mucin-type *O*-glycans, and dysregulated core fucosylation collectively influence receptor activity, ligand interaction, and downstream signaling. These glycan modifications also modulate proteolytic processing and govern cell–cell and cell–matrix interactions, thereby shaping a tumor microenvironment permissive to growth and invasion [142]. Within this context, hyper*-O*-GlcNAcylation stands out as a central integrator of metabolic and signaling cues, dynamically regulating transcription, translation, signaling pathways, metabolism, and immune interactions to promote tumor progression [28,143]. Importantly, these glycosylation changes are not mere biomarkers of transformation: they actively contribute to therapeutic resistance by altering cellular metabolism, drug uptake and distribution, and the balance between pro-survival and pro-apoptotic signaling pathways [8,144].

At the mechanistic level, accumulating evidence indicates that *O*-GlcNAcylation remodels cancer glycosylation networks by intersecting multiple regulatory layers. *O*-GlcNAcylation can modulate glycan metabolism indirectly, through epigenetic regulation and TFs networks, as well as more directly by affecting activity and stability of GTs and GHs. In colon cancer stem cells (CSCs), chromatin*-*associated *O*-GlcNAc marks co-localize with repressive histone modifications such as H3K27me3, a signature of Polycomb repressive complex 2 (PRC2)-mediated transcriptional silencing. This chromatin-level control regulates key glycosylation genes like ST3 beta-galactoside alpha-2,3-sialyltransferase 1 (ST3GAL1), a determinant of mucin-type *O*-glycan sialylation, suggesting that epigenetic *O*-GlcNAc regulation could contribute to the maintenance of stem-like phenotypes through glycosylation programs [145]. A similar transcriptional mechanism operates in differentiated cancer cells, where *O*-GlcNAc chromatin-associated TFs and cofactors (OCTFs) regulate stress-responsive glycosylation genes such as *Mannosidase alpha class 2C member 1* (*MAN2C1*), enabling adaptation to genotoxic stress [146].

Beyond transcriptional regulation, *O*-GlcNAcylation also exerts post-transcriptional effects that profoundly shape the glycosylation landscape. In HCT116 colon cancer cells, OGT depletion alters the expression of enzymes involved in *N*-glycan catabolism (Mannosidase beta, MANBA), *O*-glycan disialylation (ST8 alpha-*N*-acetyl-neuraminide alpha-2,8-sialyltransferase 6, ST8SIA6), and mucin-type *O*-glycan elongation (GALNT8 and Glucosaminyl (*N*-acetyl) transferase 3, mucin type (GCNT3)). Yet, the resulting glycomic alterations are not fully explained by transcriptional changes alone, as cell-surface α2,3-sialylation increases, β1,6-GlcNAc branching modestly rises, and the balance of sialylated versus non-sialylated GSLs shifts. These observations reveal that *O*-GlcNAcylation regulates glycosylation not only through gene expression but maybe also through direct effects on enzyme activity, intracellular trafficking, and substrate accessibility [122]. Consistently, the same study shows that OGT depletion affects both the expression and *N*-glycosylation of E-cadherin, a central regulator of epithelial–mesenchymal transition (EMT), in HT-29 colon cancer cells. Reduced β1,6-GlcNAc branching and core fucosylation correlate with enhanced localization of E-cadherin at cell–cell junctions, a feature typically associated with reinforced epithelial adhesion. Given that β1,6-GlcNAc branching is commonly linked to malignancy, these data suggest that OGT silencing may partially reverse EMT through combined transcriptional and trafficking-dependent remodeling of E-cadherin glycosylation [122].

At the enzymatic level, this multi-layered regulation is exemplified by Beta-1,4-N-acetylgalactosaminyltransferase 2 (B4GALNT2), whose high expression correlates with a favorable prognosis in colon cancer. Functionally, increased B4GALNT2 activity enhances Sd^a^ blood group determinant synthesis at the expense of sialyl Lewis x (sLe^x^), a sialylated tumor-associated carbohydrate antigen (TACA) linked to malignant progression. This glycosylation shift reshapes the tumor glycome toward a less aggressive phenotype. Beyond transcription, B4GALNT2 activity appears to be modulated by additional PTMs, including *N*-glycosylation of its luminal catalytic domain and phosphorylation and/or *O*-GlcNAcylation of the extended cytoplasmic tail of the long isoform. Such modifications may affect enzyme function, intracellular trafficking, or stability, thereby fine-tuning glycan profiles [147]. More broadly, this illustrates a recurring principle whereby *O*-GlcNAcylation intersects with other PTMs, particularly phosphorylation, to coordinately regulate enzymatic pathways and glycan structures. In colon cancer cells, PYGL provides a parallel example, where *O*-GlcNAcylation at Ser^430^ — suppressed by glucose and insulin but enhanced under glucagon signaling and hypoxia — promotes phosphorylation at Ser^15^ and enhances enzymatic activity. Such cross-regulation integrates nutrient and stress signals, linking metabolic adaptation to tumorigenesis [127]. Similarly, in bladder cancer, *O*-GlcNAcylation at Thr^229^ and Thr^233^ stabilizes the C1GalT1 by strengthening its interaction with the chaperone Core 1 β3-Gal-T-specific molecular chaperone (Cosmc) thus preventing its proteasomal degradation. This stabilization enhances T antigen synthesis, including on glycolysis-related proteins, thereby promoting glycolytic flux and supporting the pro-tumorigenic phenotype [130]. *O*-GlcNAcylation also feeds into oncogenic signaling cascades that indirectly remodel the glycome. By modulating AKT/Extracellular signal-regulated kinase (ERK) activity and Forkhead box O3 (FoxO3) stability, *O*-GlcNAcylation controls the expression of *MAN1A1*, leading to increased cell surface high-mannose *N*-glycans and promoting cholangiocarcinoma metastasis [148]. Conversely, direct *O*-GlcNAcylation of key metabolic signaling nodes such as Mechanistic target of rapamycin (mTOR) amplifies glycolytic programs and tumor progression [51,149], a process further modulated by GTs such as B3GALT5 in hepatocellular carcinoma [150]. Finally, recent work has uncovered a functional interplay between OGT and the cytosolic deglycosylase Peptide:*N*-glycanase 1 (PNG1), whereby PNG1 stabilizes OGT and, together, they regulate Nuclear factor erythroid 2–related factor 2 (Nrf2)-dependent antioxidant signaling. This axis coordinates proliferation and survival under metabolic stress, underscoring the role of glycosylation pathways as central mediators of nutrient-responsive processes in cancer and other stem cell-dependent pathologies [151].

Together, these examples position *O*-GlcNAcylation as a pivotal hub that couples metabolic reprogramming to glycosylation remodeling in cancer. By integrating nutrient availability with epigenetic regulation, enzyme activity and oncogenic signaling, *O*-GlcNAcylation shapes both the structure and function of the tumor glycome (Table 1). The multifaceted nature of these roles explains how cancer-associated glycosylation changes serve both as biomarkers and as active drivers of tumor progression, cellular plasticity, and therapeutic resistance, positioning *O*-GlcNAcylation at the core of cancer cell adaptability.

## 4 Glycosylation: a promising target for therapeutic intervention

The central role of glycosylation in coordinating metabolism, signaling and intercellular communication makes it an increasingly compelling target for therapeutic intervention across a wide spectrum of human diseases, including metabolic, cardiovascular and neurodegenerative disorders, CDGs, and cancers. Glycosylation pathways do not operate as isolated linear processes, but rather as a highly interconnected network in which changes in nucleotide sugar availability, GT activity, or *O*-GlcNAc cycling reverberate across intracellular signaling cascades and extracellular interactions alike. From this perspective, targeting glycosylation no longer appears as a collection of isolated pharmacological interventions, but instead as a coherent strategy to reshape an integrated “glyco-network” that governs cell identity and behavior. A central emerging concept is that therapeutic efficacy may lie less in the inhibition of a single enzyme or disease-associated glycan epitope than in the modulation of how UDP-GlcNAc is produced and dynamically allocated between competing glycosylation pathways. By influencing this metabolic node, it becomes possible to simultaneously impact complex glycan biosynthesis and *O*-GlcNAcylation, thereby reprogramming transcriptional networks, receptor trafficking, stress responses, and cell–matrix interactions in a coordinated manner. These network-level interventions hold the promise of resetting pathological cellular states, rather than simply inhibiting isolated downstream effects (Figure 3).

a. Targeting UDP-GlcNAc and *O*-GlcNAc Homoeostasis: From Metabolic Flux to Protein-Specific Regulation

Historically, glycosylation-targeted therapies focused on individual enzymes. GTs and GHs play key roles in controlling glycan structure, protein stability and localization. Small molecule inhibitors targeting these enzymes provided valuable mechanistic insights and demonstrated that modulating initiation, elongation, or capping steps of *N-* and *O*-glycosylation, as well as GSL and GAG synthesis, could reshape specific glycan structures and regulate protein function, cell signaling, and both cell–cell and cell–extracellular environment interactions [152,153]. However, the high interconnectivity and redundancy of glycosylation networks, together with the challenges of achieving enzyme-specific selectivity, have limited the therapeutic efficacy of strategies based on single-enzyme inhibition. This realization has prompted a shift toward network-oriented strategies, where the goal is to modulate upstream metabolic nodes that impact multiple co-dysregulated glycosylation pathways. Consistent with this perspective, early therapeutic strategies aimed to limit flux through the HBP toward UDP-GlcNAc production [17] (Figure 3 and Table 3), primarily by targeting its rate-limiting enzyme GFAT, to prevent pathological accumulation of UDP-GlcNAc and dependent-dysregulated glycosylation processes. Initial studies employing irreversible glutamine analogs such as azaserine and 6-diazo-5-oxo-l-norleucine (DON) demonstrated antitumor efficacy *in vivo* but were hampered by substantial toxicity resulting from their poor specificity toward glutamine-dependent enzymes [17,154]. These limitations have prompted the development of more selective approaches targeting GFAT and downstream components of the HBP. In this context, inhibition of PGM3 by the substrate-competitive prodrug FR054 effectively suppresses tumor growth in preclinical models, highlighting the feasibility of selectively constraining UDP-GlcNAc synthesis [155]. Complementary approaches involve mechanism-based inhibitors of UAP1, including α,β-methylenebisphosphonate UTP (meUTP) [156] and the drug-like fragment GAL-012 [157], as well as targeting the salvage pathway — such as N-acetylglucosamine kinase (NAGK) inhibition with 3-O-methyl-N-acetyl-d-glucosamine [158]. Together, these approaches expand the therapeutic landscape for fine-tuning glycosylation flux. Nevertheless, whether such strategies can achieve durable efficacy with acceptable safety profiles *in vivo* remains an important question for future investigations. Conversely, in diseases with UDP-GlcNAc deficiency, straightforward strategy is direct supplementation with D-GlcNAc or D-GlcNH_2_, which are metabolized into the HBP (Figure 3 and Table 3). *In vivo,* oral D-GlcNAc supplementation effectively restores *N*-glycan branching on hepatic glycoproteins, enhancing key receptors and transporters stability, nutrient absorption, glycogen and lipid storage, as demonstrated in *Mgat5*^*−*/−^ mice [42]. Similarly, in a *GFAT1* deficiency T cell model of impaired *de novo* HBP, dietary D-GlcNH_2_ supplementation partially restored UDP-GlcNAc levels and rescued glycosylation-dependent T cell development, supporting the capacity of HBP precursors to replenish UDP-GlcNAc *in vivo* [159].

**Table 3:** Therapeutic and experimental strategies for modulating UDP-GlcNAc availability, *O*-GlcNAc homeostasis, and glycan-directed targeting.

Strategy	Target	Representative compounds	Mechanism of action	Key features & limitations	References
Strategies for regulating HBP flux and *O*-GlcNAc homeostasis					
**HBP flux inhibition (UDP-GlcNAc pool reduction)**	GFAT (rate-limiting), PGM3, UAP1, NAGK	GFAT: Azaserine, DON; PGM3: FR054; UAP1: meUTP, GAL-012; NAGK: 3-O-methyl-N-acetyl-d-glucosamine	Competitive substrate analogues (azaserine, DON, FR054, meUTP, 3-O-methyl-N-acetyl-d-glucosamine) or active-site-directed (GAL-012) inhibition of *de novo* and salvage HBP enzymes	Modest potency, lack or limited specificity (azaserine and DON target multiple glutamine amidotransferases; meUTP likely inhibits UAP1 and other UTP-dependent enzymes; GAL-012 acts as a multi-UDP-hexose pyrophosphorylases inhibitor (e.g. UAP1, UGP2 and GALT); 3-O-methyl-N-acetyl-d-glucosamine inhibits both NAGK and GNE); mostly preclinical tools; FR054 is the most selective PGM3 inhibitor with *in vivo* activity	[17,[154], [155], [156], [157], [158]]
**HBP metabolic supplementation (UDP-GlcNAc pool expansion)**	HBP precursors	D-GlcNAc, D-GlcNH_2_	Metabolic incorporation into HBP pathway	Simple strategy; oral supplementation may increase UDP-GlcNAc levels and support glycosylation *in vitro* and *in vivo*	[42,159]
**OGT inhibition (reduced *O*-GlcNAcylation)**	OGT	Metabolic analogues: Alloxan, UDP-5S-GlcNAc, Ac_4_-5S-GlcNAc, 5S-GlcNHex	Direct competition at UDP-GlcNAc binding site (alloxan; UDP-5S-GlcNAc) or metabolic conversion *via* the HBP salvage pathway into inhibitory UDP-GlcNAc analogues (Ac_4_-5S-GlcNAc; 5S-GlcNHex)	Variable stability, solubility and cell permeability; reported (for alloxan) and potential off-target inhibition of other glycosyltransferases; Ac_4_-5S-GlcNAc is cell-permeable and widely used in preclinical studies but limited *in vivo* by poor aqueous solubility; the optimized amphiphilic derivative 5S-GlcNHex is suitable for *in vivo* administration	[[160], [161], [162], [163], [164], [165], [166]]
		Small molecules: Goblin1-2; BZX1/2; ST045849; OSMI-1–4; L01	Direct binding to OGT catalytic site *via* bisubstrate-mimetic (Goblin), covalent (BZX), and non-covalent small-molecule inhibition (OSMI, ST045849, L01)	Improved selectivity compared to metabolic analogues; some compounds show good potency, cell permeability and *in vivo* activity (e.g., OSMI; L01); clinical translation remains under investigation	[29,[167], [168], [169], [170], [171], [172], [173], [174], [175], [176], [177], [178]]
**OGA inhibition (increased *O*-GlcNAcylation)**	OGA	Metabolic analogues: PUGNAc; STZ, GlcNAcstatins	Active-site occupation by GlcNAc mimetics	High potency but variable selectivity; early compounds (STZ, PUGNAc) inhibit lysosomal hexosaminidases (HEXA/B), whereas GlcNAcstatins show improved selectivity and potency but limited drug-like properties	[[180], [181], [182]]
		Small molecules: NButGT; Thiamet-G	Oxazoline transition-state mimetics targeting OGA catalytic mechanism	High potency and selectivity toward OGA over HEXA/B; NButGT shows limited stability in aqueous solution, whereas cell-permeable Thiamet-G displays high aqueous stability, is bioavailable, crosses the BBB, and is widely used *in vitro* and *in vivo*	[183]
		Next-generation small molecules: MK-8719; LSN3316612; ASN90 (FNP-223); ASN51; LY3372689; BIIB113	Highly optimized, selective OGA inhibitors with drug-like properties and BBB penetration	Clinical evaluation in tauopathies; FNP-223 retains FDA Fast Track designation (2025) and is ongoing in phase II for PSP (NCT06355531), while radiolabeled LSN3316612 showed successful phase I PET imaging in healthy volunteers (NCT03632226); other programs were discontinued after phase I (MK-8719, BIIB113) or phase II in AD (ASN51, LY3372689), highlighting translational challenges despite strong target engagement	[[183], [184], [185], [186], [187], [188], [189], [190], [191], [192], [193], [194], [195]]
**Splicing modulation (indirect *O*-GlcNAc regulation)**	OGT, OGA	GSK690693, Y-33075, OTS964, indisulam, GNF2133	Modulation of alternative splicing leading to reduced OGT and/or OGA expression	Indirect strategy that alters *O*-GlcNAc homeostasis without direct enzyme inhibition; limited selectivity, broad transcriptome-wide effects, and pleiotropic mechanisms constrain the characterization and optimization of these splicing modulators for therapeutic applications	[179]
**Substrate-selective *O*-GlcNAc modulation strategies**	OGT, OGA, specific *O*-GlcNAcylated target protein	Nanobody-OGT/OGA fusion systems; dual-specificity aptamers; OGTACs	Substrate- and proximity-induced recruitment of OGT/OGA to specific proteins	Promising proof-of-concept tools for selective *O*-GlcNAc editing in living cells, with distinct targetability profiles (endogenous targets for aptamers; exogenous tagged targets for nanobody-OGT/OGA systems; and currently for OGTACs), but requiring further optimization of endogenous target engagement and delivery, as well as *in vivo* validation	[152,[197], [198], [199]]
**Exploitation of disease-associated glycan signatures for selective targeting and drug delivery**					
**Glycoconjugate-based selective targeting and drug delivery**	Disease-associated cell-surface glycan signatures	Glycan-based vaccines (e.g., TACA-directed vaccines), glycoconjugates (e.g., glucose-conjugated small molecules), glycan-directed carriers (e.g., hyaluronic acid-CD44, fucoidan-P-selectin, lectin-glycan targeting systems)	Exploitation of altered glycosylation as biomarkers and targeting ligands for selective delivery or immune recognition	Enables selective diagnosis, patient stratification, and targeted delivery across multiple diseases, with improved specificity and reduced systemic toxicity potential; glycan heterogeneity and context dependence guide further optimization for clinical translation	[142,[200], [201], [202], [203],[210], [211], [212],^214^]

This table summarizes the main pharmacological and molecular strategies used to modulate intracellular UDP-GlcNAc availability, *O*-GlcNAc homeostasis and glycan-mediated disease targeting. These strategies include inhibition or supplementation of hexosamine biosynthetic pathway (HBP) flux, direct or indirect modulation of *O*-GlcNAc transferase (OGT) and *O*-GlcNAcase (OGA) enzymes, including splicing-based regulatory approaches, substrate-selective *O*-GlcNAc editing technologies, and glycan-based targeting strategies for selective therapeutic delivery. For each approach, representative compounds, mechanisms of action, and key features or limitations are indicated, highlighting current advances and challenges for preclinical and clinical translation.

5S-GlcNHex, 2-deoxy-2-N-hexanamide-5-thio-d-glucopyranoside; Ac_4_-5S-GlcNAc, 2-acetamido-1,3,4,6-tetra-*O*-acetyl-2-deoxy-5-thio-α-d-glucopyranose; AD, Alzheimer's disease; BBB, blood–brain barrier; BZX, benzoxazolinone derivatives; CD44, Cluster of differentiation 44; DON, 6-diazo-5-oxo-l-norleucine; FDA, Food and Drug Administration; GFAT, Glutamine:fructose-6-phosphate amidotransferase; GlcNAc, N-acetylglucosamine; GlcNH_2_, glucosamine; GNE, Glucosamine (UDP-N-acetyl)-2-epimerase/N-acetylmannosamine kinase; HEX, hexosaminidase; meUTP, α,β-methylenebisphosphonate UTP; NAGK, N-acetylglucosamine kinase; *O*-GlcNAc, *O*-linked N-acetylglucosamine; OGA, *O*-GlcNAcase; OGT, *O*-GlcNAc transferase; OGTAC, OGT-targeting chimera; PGM3, Phosphoglucomutase 3; PET, positron emission tomography; PSP, progressive supranuclear palsy; STZ, streptozotocin; TACA, tumor-associated carbohydrate antigen; UAP1, UDP-N-acetylglucosamine pyrophosphorylase 1; UDP-5S-GlcNAc, uridine diphospho-5-thio-*N*-acetylglucosamine; UDP-GlcNAc, uridine diphosphate N-acetylglucosamine; UGP2, UDP-glucose pyrophosphorylase 2.

Beyond metabolic interventions, targeting dynamic *O*-GlcNAcylation represents an integrative approach to modulate glycosylation networks with downstream effects on transcriptional programs and metabolic pathways [27]. In this context, multiple pharmacological approaches have been developed to inhibit OGT and reduce global protein *O*-GlcNAcylation [27] (Figure 3 and Table 3). Early efforts relied on substrate analogs such as alloxan, which can occupy the UDP-GlcNAc binding site but suffer from poor stability, limited specificity, and cellular toxicity [[160], [161], [162], [163]]. To overcome these limitations, alternative strategies have leveraged HBP, notably through diphospho-5-thio-N-acetylglucosamine (UDP-5S-GlcNAc) [164] and more lipophilic, cell-permeable precursors 2-acetamido-1,3,4,6-tetra-*O*-acetyl-2-deoxy-5-thio-α-d-glucopyranose (Ac_4_-5S-GlcNAc) [165] 2-deoxy-2-N-hexanamide-5-thio-d-glucopyranoside (5S-GlcNHex) [166]. More advanced bisubstrate inhibitors, such as Goblin1 and Goblin2, covalently bridge donor and acceptor moieties to block catalysis but their poor cellular permeability currently limits their use to *in vitro* applications [167]. In parallel, high-throughput screening (HTS) approaches have identified several small-molecule OGT inhibitors, including ST045849 [168], benzoxazolinone (BZX) derivatives [169], L01 [170], and the OSMI series (OSMI-1 to −4) [[171], [172], [173]]. Among these, OSMI compounds have become widely used in mechanistic and preclinical studies owing to their favorable physicochemical properties, cellular permeability, and comparatively limited off-target toxicity. Preclinical *in vivo* studies demonstrate that OSMI compounds can modulate disease-relevant pathways across multiple contexts. OSMI-1 reduces tumor growth and enhances apoptosis in hepatocarcinoma (HCC) and colon cancer xenograft models [174,175]. Beyond oncology, OSMI-1 exerts broad anti-inflammatory and anti-fibrotic effects: it reshapes immune responses under both systemic and tissue-specific inflammatory stress [176,177], and attenuates myofibroblast activation and pro-fibrotic gene expression in liver injury models [29]. In the nervous system of Rett syndrome mouse model, systemic administration of OSMI-1 restores hippocampal *O*-GlcNAcylation and improves recognition memory, redox balance, and mitochondrial function [178]. Beyond direct enzymatic inhibition, recent work has uncovered an additional layer of regulation through splicing modulation. Several compounds originally developed as kinase inhibitors or splicing modulators — including GSK690693, Y-33075, OTS964, indisulam, and GNF2133 — were shown to downregulate both *OGT* and *OGA* expression *via* distinct alternative splicing mechanisms. This unexpected convergence highlights a novel strategy to perturb *O*-GlcNAc homeostasis at the level of gene regulation, rather than through enzyme catalytic activity, thereby expanding the pharmacological toolbox beyond the conventional targeting of *O*-GlcNAc cycling enzymes [179].

Potent and selective inhibitors have also been developed for OGA, the *O*-GlcNAc hydrolase that promotes dynamic cycling of the *O*-GlcNAcylation [27] (Figure 3 and Table 3). Early GlcNAc analogs streptozotocine (STZ) [180] and PUGNAc [181] were instrumental in establishing proof of principle but suffered from limited selectivity and off-target effects, which restricted their translational relevance. These limitations spurred the rational design of more selective OGA inhibitors, such as GlcNAcstatin [182] and oxazoline-based compounds including NButGT and Thiamet-G [183], and next-generation MK-8719 [184] and LSN3316612 [185,186]. Thiamet-G represented a major conceptual advance due to its ability to cross the blood–brain barrier, allowing *in vivo* modulation of *O*-GlcNAcylation within the central nervous system. MK-8719 and LSN3316612 further emerged as optimized clinical candidates, combining high nanomolar potency, excellent selectivity toward OGA, and favourable drug-like properties. Building on these foundational scaffolds, additional OGA inhibitors have been generated through HTS and medicinal chemistry optimization, including ASN51 [187], FNP-223 (formerly ASN90) [188], ceperognastat (LY3372689) [189], and BIIB113 [190]. Collectively, these compounds have catalysed extensive preclinical and clinical exploration, particularly in the field of neurodegenerative diseases. Evidence that human tau is *O*-GlcNAcylated, together with the finding that pathological tau aggregates in AD brains are largely devoid of this modification, provided a strong mechanistic rationale for targeting OGA. Proof-of-concept studies using Thiamet-G in rodent models demonstrated that acute elevation of global *O*-GlcNAc levels reduces site-specific tau phosphorylation, thereby establishing OGA inhibition as a viable therapeutic strategy [183]. This concept has since progressed toward clinical translation, with several OGA inhibitors advancing through clinical trials at different stages for AD, progressive supranuclear palsy (PSP), and related tauopathies [187,[190], [191], [192], [193]]. Beyond therapeutic applications, OGA inhibitors have also been repurposed as molecular imaging tools. Radiolabeled derivatives of ASN51 [194] and ceperognastat [195] have been developed for positron emission tomography (PET), enabling quantitative assessment of brain OGA occupancy *in vivo* and providing critical pharmacodynamic readouts in both preclinical models and humans. Notably, FNP-223 received Fast Track designation from the U.S. Food and Drug Administration (FDA) in 2025 for the treatment of PSP, underscoring the growing clinical momentum of this pharmacological class. Importantly, the therapeutic scope of OGA inhibition is likely not restricted to neurodegeneration. Given that dysregulated hypo-*O*-GlcNAcylation is a hallmark of several genetic and acquired disorders, OGA inhibitors may hold promise in other pathological contexts, including OGT-CDG. In a *Drosophila* model carrying the OGT-CDG–associated C941Y mutation, characterized by impaired synaptogenesis and reduced sleep stability, pharmacological inhibition of OGA with Thiamet-G restored global *O*-GlcNAcylation and partially rescued synaptic defects at the neuromuscular junction, as well as sleep phenotypes in adult flies [196].

Despite the therapeutic promise of targeting *O*-GlcNAc cycling *via* OGT or OGA, the ubiquitous and essential nature of this PTM presents challenges due to off-target effects on critical proteins. These limitations underscore the need for advanced targeting approaches that extend beyond global modulation of *O*-GlcNAcylation. In this context, strategies enabling selective manipulation of *O*-GlcNAc levels in defined cellular states or activated cell populations may help mitigate systemic toxicity. Moreover, innovative tools — including substrate-selective nanobody-OGT [197] or OGA [198] fusion systems, dual-specificity aptamers [199], and *O*-GlcNAcylation targeting chimeras (OGTACs) [152] — allow precise writing or erasing *O*-GlcNAcylation on individual proteins — potentially disease-relevant glycosylation-related proteins — emerging as a new frontier for therapeutic intervention (Figure 3 and Table 3).

b. Diseases-Associated Glycan Signatures for Diagnosis and Therapeutic Targeting

While targeted modulation of HBP or *O*-GlcNAc cycling offers precise control over protein-specific glycosylation, a complementary approach capitalizes on the glycan alterations that naturally arise in diseases (Figure 3 and Table 3). Pathological remodeling of the glycome generates distinct molecular patterns that differentiate affected cells from their healthy counterparts, providing both diagnostic markers and selective vulnerabilities that can be exploited for therapeutic intervention [142]. In cancer, this principle is exemplified by tumor-associated carbohydrate antigens (TACAs), such as Globo-H, monosialoganglioside 2 (GM2), Thomsen-Friedenreich (TF), Tn, STn, and Le^y^, which have been extensively leveraged for therapeutic targeting. These TACAs have formed the basis of glycan-based vaccines that, in many cases, have progressed from early prototypes to phase I clinical trials [200], demonstrating how aberrant surface glycans can be harnessed to elicit tumor-specific immune responses and guide cancer immunotherapy. Aberrant glycosylation also provides opportunities to enhance the selectivity and efficacy of drug therapies. By conjugating chemotherapeutic agents to monosaccharide or glycan motifs, glycoconjugate drugs improve uptake by diseased cells while limiting systemic toxicity [142,201]. Similarly, glycan-directed nanotherapies exploit these pathological signatures to enable targeted delivery through nanoparticles functionalized with glycan-binding ligands or antibodies [202]. For instance, a hyaluronic acid-based nanocarrier co-encapsulating doxorubicin and cisplatin was engineered to target CD44^+^ breast cancer cells, resulting in superior drug distribution within tumor and reduced off-target effects, demonstrating the potential of such platforms to improve both the safety and efficacy of chemotherapy [203].

Although these approaches have been most extensively developed in oncology, they illustrate a broader therapeutic paradigm in which disease-associated glycan signatures can be exploited for diagnosis and selective targeting of other diseases characterized by altered glycosylation (Figure 3 and Table 3). In neurodegenerative disorders, changes in *N*-glycosylation profile of amyloid-β [204] or transferrin [205] in cerebrospinal fluid and blood have been proposed as highly specific indicators of AD, PD and other neurodegenerative diseases. In metabolic diseases, where nutrient excess and metabolic stress reshape the glycome, carbohydrate antigen 19-9 (CA19-9) — a cancer-associated glycan epitope — correlates with insulin resistance and glycemic and lipid dysregulation in patients with obesity and T2D undergoing rapid metabolic normalization after Roux-en-Y gastric bypass [206]. These observations highlight how glycan biomarkers can capture dynamic changes in metabolic state and glycolipid toxicity, further underscoring the diagnostic value of pathological glycosylation. In the cardiovascular context, glycosylation of von Willebrand factor (vWF), a central regulator of hemostasis, may exacerbate the pro-thrombotic environment characteristic of diabetes by altering its synthesis, secretion, structural stability, proteolysis and haemostatic function [207]. Mapping site-specific vWF glycosylation and validating these modifications in patient samples could therefore identify individuals at increased risk of macrovascular complications, while revealing new opportunities for therapeutic intervention [208]. Interestingly, vWF with reduced sialylation in circulating extracellular vesicles has emerged as a biomarker of major depressive disorder [209].

The identification of disease-specific glycan signatures reinforces the rationale for glycan-directed targeting strategies beyond oncology. Proof-of-concept studies show that cell surface glycan receptors and the glycocalyx can be exploited to guide nanomaterials toward specific cell populations. In the central nervous system, lectin-conjugated nanodiamonds selectively target astrocytes, microglia, and neurons *via* surface glycan receptors, exhibiting cell-type-specific uptake profiles under physiological conditions. Moreover, appropriate surface functionalization, including polyethylene glycol and lectins such as wheat germ agglutinin (WGA), may facilitate blood–brain barrier crossing without compromising its integrity [210]. Glucose-conjugated small molecules exploiting GLUT1 overexpression and HBP-dependent glycosylation were first explored in cancer cells to enhance the delivery of cytotoxic drugs (e.g., chlorambucil, methane sulfonate, paclitaxel, azomycin, adriamycin, oxaliplatin) [211]. More recently, same conjugated antipsoriatic agents (cyclosporine, acitretin, and tofacitinib) were used to selectively target immune and epidermal cells in psoriasis inflamed tissue [212]. This approach could provide a broader strategy for metabolic diseases in which GLUT1 activity is elevated in specific organs, including the liver [213]. In cardiovascular disease, glycan-based materials such as fucoidan — a highly sulfated l-fucose-containing polysaccharide that mimics P-selectin glycoprotein ligand-1 (PSGL-1) — exploit P-selectin–rich thrombi to locally deliver antithrombotic and cardioprotective agents, concentrating therapy at vascular injury sites while limiting systemic exposure [214]. Aberrant glycosylation in CDGs can limit the availability of glycan motifs necessary for adeno-associated virus (AAV) binding and lower specific coreceptors levels, highlighting a potential obstacle for effective gene delivery. These findings underscore the importance of understanding and adapting viral vectors for efficient transduction in contexts of altered protein glycosylation, including CDGs and neurodegenerative diseases [215]. Collectively, these examples illustrate a unified therapeutic paradigm in which disease-associated glycan signatures serve both as biomarkers that guide diagnosis, patient stratification, and therapeutic response prediction, and as entry points for selective targeting across cancer, metabolic, cardiovascular, neurodegenerative diseases, and CDGs (Figure 3 and Table 3).

## 5 Conclusion

Glycosylation has long been approached through discrete biochemical categories, largely defined by subcellular localization, enzymatic machinery, and presumed stability of the modification. The body of evidence discussed in this review challenges this compartmentalized view and supports a paradigm shift toward an integrated, network-based understanding of glycosylation. Within this framework, complex luminal glycosylations and *O*-GlcNAcylation are not independent layers of regulation, but interdependent processes coordinated by shared metabolic resources and regulatory feedback loops. Central to this network is UDP-GlcNAc, whose abundance and intracellular distribution reflect cellular nutrient status and metabolic flux through the HBP. Variations in UDP-GlcNAc availability do not selectively affect individual glycosylation pathways; rather, they propagate across the glycosylation landscape, reshaping *N*- and *O*-glycosylation, GAG and glycolipid synthesis, as well as nucleocytoplasmic *O*-GlcNAcylation. In this way, UDP-GlcNAc functions as a metabolic hub that couples cellular metabolism to both intracellular signaling and extracellular architecture. Within this interconnected system, *O*-GlcNAcylation emerges as a supplementary and highly adaptive regulatory layer. By dynamically modifying chromatin regulators, TFs, metabolic enzymes and components of the glycosylation machinery itself, *O*-GlcNAcylation enables rapid tuning of an otherwise structurally stable glycome. it operates as a responsive interface that both senses and modulates broader changes in glycosylation flux and network organization. Importantly, this network perspective has profound implications for human diseases. Pathological phenotypes associated with metabolic disorders, cancer, neurodegeneration, cardiovascular diseases, or CDGs can no longer be interpreted as isolated defects in single pathways. Instead, they reflect systemic rewiring of glycosylation networks driven by metabolic imbalance, altered UDP-GlcNAc flux, leading to coordinated deregulation of multiple UDP-GlcNAc–dependent glycosylation processes. This framework provides a coherent explanation for why perturbations in one glycosylation process frequently propagate across the system, manifesting as broad and pleiotropic cellular dysfunctions**.** From a therapeutic standpoint, embracing glycosylation as a regulatory network opens new avenues beyond global inhibition or supplementation strategies. Targeting UDP-GlcNAc metabolism or *O*-GlcNAcylation offers the advantage of rebalancing glycosylation fluxes across interconnected pathways, rather than correcting individual modifications in isolation. By modulating shared metabolic and regulatory hubs, such approaches may restore coordinated glycosylation programs while preserving essential cellular functions. Future therapeutic strategies will likely benefit from combining metabolic intervention, protein-specific glycosylation editing, and glycan-based targeting, moving toward a more nuanced and systems-level manipulation of the glycosylation landscape (Figure 3). Understanding and harnessing this network will be essential for the development of next-generation diagnostic and therapeutic strategies.

## CRediT authorship contribution statement

**Ninon Very:** Writing – original draft, Conceptualization. **Ikram El Yazidi-Belkoura:** Writing – original draft, Supervision, Conce-ptualization.

## Declaration of competing interest

The authors declare that they have no known competing financial interests or personal relationships that could have appeared to influence the work reported in this paper.

## Data Availability

No data was used for the research described in the article.
